# Transmission of α-synucleinopathy from olfactory structures deep into the temporal lobe

**DOI:** 10.1186/s13024-016-0113-4

**Published:** 2016-06-30

**Authors:** Daniel M. Mason, Negin Nouraei, Deepti B. Pant, Kristin M. Miner, Daniel F. Hutchison, Kelvin C. Luk, John F. Stolz, Rehana K. Leak

**Affiliations:** Division of Pharmaceutical Sciences, Duquesne University, 600 Forbes Ave, Pittsburgh, PA 15282 USA; Department of Pathology, University of Pennsylvania, Philadelphia, PA 19147 USA; Department of Biological Sciences, Duquesne University, Pittsburgh, PA 15282 USA

**Keywords:** Hippocampus, Olfaction, Parkinson’s disease, Aging, Synuclein, Lewy, Smell

## Abstract

**Background:**

α-synucleinopathy emerges quite early in olfactory structures such as the olfactory bulb and anterior olfactory nucleus (OB/AON) in Parkinson's disease. This may contribute to smell impairments years before the commencement of motor symptoms. We tested whether α-synucleinopathy can spread from the OB/AON to regions of the limbic telencephalon that harbor connections with olfactory structures.

**Findings:**

α-synuclein fibrils were infused into the OB/AON. Inclusions containing pathologically phosphorylated α-synuclein (pSer129) were observed three months later in the piriform and entorhinal cortices, amygdala, and hippocampal formation. The retrograde tract-tracer FluoroGold confirmed the existence of first-order afferents at these sites. Some sites harbored FluoroGold^+^ neurons but no inclusions, suggestive of selective vulnerabilities. Multiple areas close to the injection site but not connected with the OB/AON remained free of inclusions, suggesting a lack of widespread uptake of fibrils from interstitial diffusion. Two independent pSer129 antibodies revealed the same labeling patterns and preadsorption control experiments confirmed a loss of pSer129 staining. Dense total α-synuclein (but not pSer129) staining was apparent in the OB/AON 1.5 h following fibril infusions, suggesting that pSer129^+^ staining did not reflect exogenously infused material. Waterbath sonication of fibrils for 1 h improved α-synucleinopathy transmission relative to 1 min-long probe sonication. Electron microscopy revealed that longer sonication durations reduced fibril size. The Thioflavin stain labeled cells at the infusion site and some, but not all inclusions contained ubiquitin. Three-dimensional confocal analyses revealed that many inclusions ensconced NeuN^+^ neuronal nuclei. Young and aged mice exhibited similar topographical spread of α-synucleinopathy.

**Conclusions:**

1) α-synucleinopathy in this model is transmitted through some, but not all neuroanatomical connections, 2) pathology is largely confined to first-order afferent sites at three months and this is most parsimoniously explained by retrograde transport, and 3) transmission in aged animals is largely similar to that in young control animals at three months post-infusion.

**Electronic supplementary material:**

The online version of this article (doi:10.1186/s13024-016-0113-4) contains supplementary material, which is available to authorized users.

## Introduction

Due to the unique anatomical position of the olfactory bulb (OB), this structure may be exposed to higher concentrations of inhaled environmental toxins and pathogens than deeper brain structures. Perhaps as a result of these exposures, olfactory dysfunction is an early feature of many neurodegenerative conditions [1]. For example, approximately 90 % of Parkinson’s patients experience smell disruption, which may affect their ability to detect decomposing food and hazardous fumes [2]. Some of the first brain regions to develop α-synuclein^+^ Lewy pathology in this condition are the OB and adjoining anterior olfactory nucleus (AON), well before the emergence of Lewy pathology in deeper mesencephalic structures [3–5]. α-synucleinopathy has recently been speculated to spread from cell to cell across neuroanatomical circuits, with the medulla oblongata as the potential “port of entry” into the brain [3, 6–10]. Although α-synucleinopathy is not thought to expand significantly into the brain from olfactory structures compared to more extensive transmission from vagal/medullary nodes [3], if Lewy pathology does spread through neuroanatomical circuits, its emergence in olfactory areas such as the entorhinal allocortex might serve as an important gateway into the hippocampal archicortex. Lewy pathology in the hippocampus and entorhinal cortex has profound implications for cognitive dysfunction, which a sizeable fraction of patients with Parkinson’s disease or Lewy body disease eventually develop [11, 12]. Thus, the major goal of the present study was to determine whether α-synucleinopathy could spread from the OB/AON into limbic structures of the temporal lobe in non-transgenic mice that do not overexpress α-synuclein.

Rey and colleagues demonstrated that human α-synuclein oligomers and monomers, but not fibrils, are taken up following infusions into the OB and rapidly transported to the AON and frontal and piriform cortices within hours, but not to the entorhinal cortex or hippocampus [13]. However, Lewy-like pathology was not demonstrated in this short-term study. In an edifying review article, Rey presented pilot data showing long-term transmission of α-synucleinopathy from the OB into the entorhinal cortex, amygdala, and hippocampal formation [14]. However, the pathology was only shown at higher magnification and appeared sparse, perhaps because the α-synuclein injections were centered in the rostral extent of the OB, distant from the AON [13, 14]. The AON is a major site of olfactory pathology in Parkinson’s disease [3, 4, 15, 16]. Kordower and colleagues reported age-related changes in α-synuclein expression that may influence the development of Lewy pathology [17, 18]. Therefore, we sought to develop a robust model of α-synucleinopathy in the rhinencephalon of young and aged mice by infusing preformed α-synuclein fibrils into the OB/AON.

Fibril sonication parameters are important in the transmission of pathology [19]. Therefore, in the first study we sonicated recombinant, wildtype α-synuclein fibrils of mouse origin for 1 min with a probe or for 1 h in an inexpensive waterbath sonicator prior to infusion in the OB/AON of two month-old mice. Three months later, sagittal brain sections were collected and stained with a) the amyloid stain Thioflavin S, b) two antibodies against pathologically phosphorylated α-synuclein (pSer129), and c) two antibodies against ubiquitin—all established markers of Lewy-like pathology [20–23]. A separate cohort of mice was sacrificed 1.5 h following vehicle or fibril infusions to visualize the center of the injection site, as this timepoint shows the highest transfer of human α-synuclein following OB injections according to Rey and colleagues [13]. In the second study, we infused the retrograde tract-tracer FluoroGold into the OB/AON in order to a) determine if the sites that developed α-synucleinopathy in Study 1 project directly into the OB/AON and b) again confirm the center of the injection site at the stereotaxic coordinates employed in the first study. As the first study revealed that 1 h waterbath sonication was more effective in eliciting pathology than 1 min-long probe sonication, we performed a third study with fibrils sonicated for 1 h or 24 h in a waterbath. In order to confirm that the waterbath sonication protocol would also work in a different set of neuroanatomical circuits, we infused the CA2/CA3 fields of the hippocampus in the third study. In previous studies employing preformed α-synuclein fibrils, the fibrils were also infused into the hippocampus, among other sites [6, 24]. CA2/CA3 were chosen as the target site of infusion in the present study because they are the major hippocampal fields to exhibit α-synucleinopathy in Parkinson’s disease or diffuse Lewy body disease [3, 25, 26]. Finally, in order to ensure that the transmission of α-synucleinopathy through the limbic rhinencephalon from the OB/AON was reproducible, we performed a fourth study infusing fibrils into the OB/AON of older animals (5 and 17 months of age). These experiments also allowed us to determine if α-synucleinopathy transmission would be more extensive with age-related loss of protein quality control.

## Methods

For sonication protocols, injection parameters, animal numbers, statistics, etc., please see the detailed Methods section in Additional file 1. All procedures were approved by the Duquesne University IACUC (protocol 1403–03) and in compliance with the *NIH Guide*. For the first study, phosphate-buffered saline (PBS) or α-synuclein fibrils (5 μg in 1 μL PBS; sonicated for 1 min with a probe or 1 h in a waterbath) were unilaterally infused into the right OB/AON of non-transgenic male CD1 mice at two months of age. Sagittal brain sections were collected three months later and stained with 1) Thioflavin S, 2) an antibody against the specific neuronal nuclear marker NeuN, 3) two antibodies against pSer129, and 4) two antibodies against ubiquitin (see Additional file 1: Table S3). A separate cohort of animals was sacrificed 1.5 h post-infusion to identify the center of the injection site and the extent of fibril diffusion. For the second study, two month-old mice were infused with the retrograde tracer FluoroGold into the OB/AON to label afferent neurons that send direct projections to the site of infusion and to again confirm the center of the injection site at the same stereotaxic coordinates as in the first study. For the third study, mice were infused with PBS or fibrils (5 μg/1 μL; sonicated for 1 h or 24 h in a waterbath) into hippocampal CA2/CA3 and sacrificed three months later, to ensure that the waterbath sonication protocol worked in a different set of neuroanatomical circuits and to determine if 24 h sonication worked even better than 1 h sonication. For the fourth study, older mice (5 and 17 months) were infused with PBS or fibrils (5 μg/1 μL; 1 h waterbath-sonicated) into the OB/AON to ensure reproducibility of α-synucleinopathy transmission from olfactory structures and to determine if there was spread to additional brain regions in aged 17 month-old animals.

## Results and discussion

### Study 1: Transmission of α-synucleinopathy through the limbic rhinencephalon from the OB/AON

**Fig. 1:**
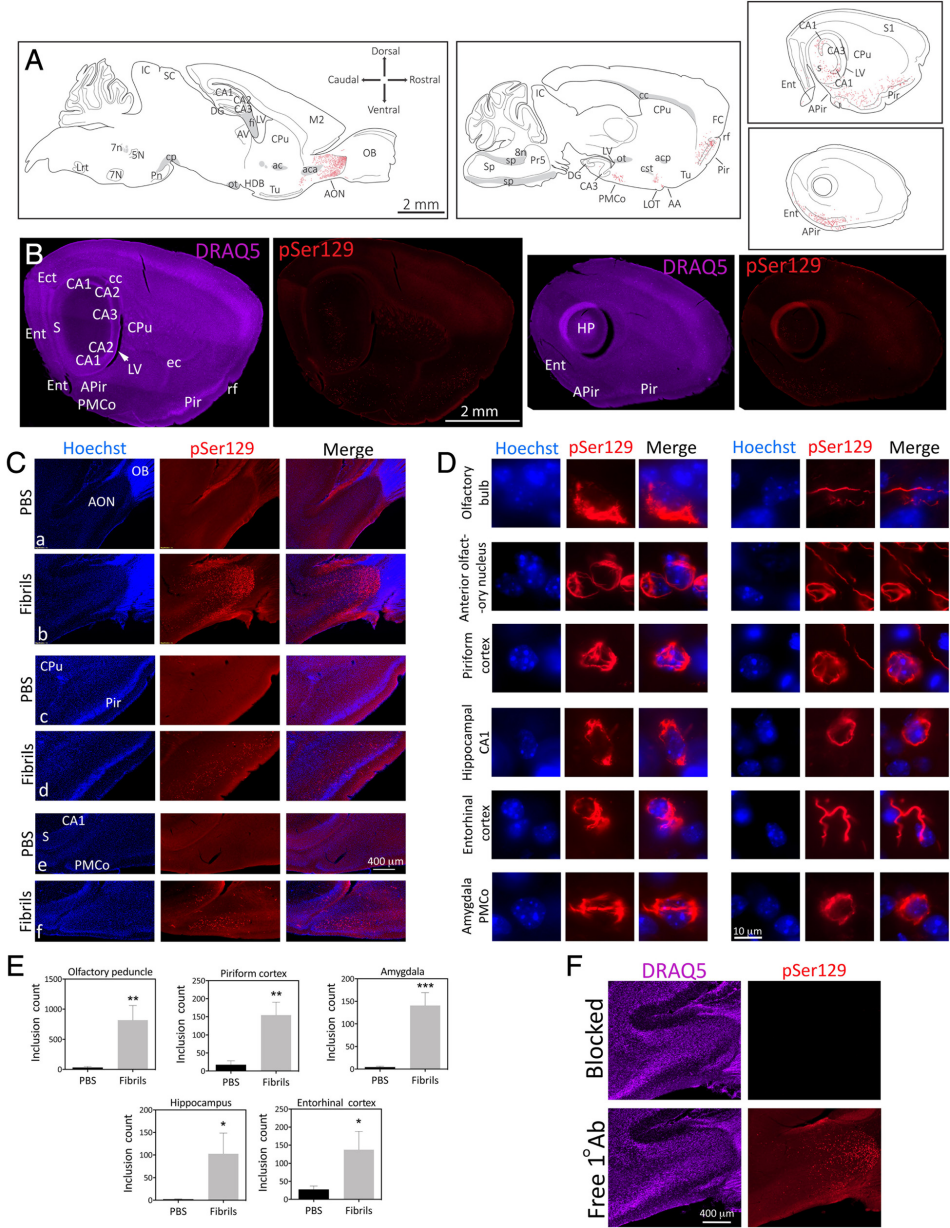
α-synucleinopathy is transmitted from the OB/AON to deeper rhinencephalic structures. Two month-old CD1 mice were unilaterally infused with α-synuclein fibrils (5 μg) or an equal volume of phosphate-buffered saline (PBS) into the olfactory bulb and adjoining anterior olfactory nucleus (OB/AON). Fibrils were sonicated for 1 h in a waterbath prior to infusion. Three months later, sagittal brain sections were collected and immunostained for pathologically phosphorylated α-synuclein (pSer129; red). Fibril and PBS groups were stained and photographed in parallel. **a** Large, high-quality microscopic photomontages of pSer129 and nuclear labeling were stitched together and viewed with Adobe Illustrator software on a tablet. Sagittal schematics of only obvious and clearly visible brain cytoarchitectonics (solid lines), myelinated fiber bundles (gray shading), pSer129^+^ neurites (red flourishes), and pSer129^+^ somal inclusions (red dots) were then traced with the pencil and paintbrush tools. All abbreviations are listed in Additional file 1: Table S1. **b** Examples of stitched photomontages of pSer129 immunostaining and DRAQ5 nuclear labeling following fibril infusions in the OB/AON (different sections than drawn in A). Please download the high-resolution supplemental versions of these files at thelink at the end of this article (Additional file 3) or email the corresponding author at leakr@duq.edu for access to the files and zoom in and out of the stitched montages in order to appreciate the density of the pathology, distinguish it from background, and judge the precise anatomical location. **c** pSer129 and nuclear Hoechst staining in the olfactory peduncle (*a-b*), the piriform cortex (*c-d*), and the hippocampus and amygdala (*e-f*) in PBS and fibril-infused animals. Abbreviations in Additional file 1: Table S1. **d** Images of pSer129^+^ inclusions and Hoechst-stained nuclei were captured using a 100× oil objective. The pSer129^+^ inclusions were perinuclear or found in processes (also see confocal images in Fig. 2b and Additional file 2, which shows two 3D-like videos of pSer129/NeuN labeled cells). The pSer129^+^ cell in the OB (uppermost row) was in the mitral cell layer, which is known to house large somata. Images of the sparse label in the OB can be viewed in Additional file 1: Figure S1, S2, and the images below Additional file 1: Table S2. **e** pSer129^+^ inclusion numbers were counted in ImageJ software by a blinded observer, after the same threshold values were applied across all images. Inclusion counts were 29.8 ± 20.2 (mean ± SEM) for PBS mice and 817.0 ± 242.5 for fibril mice in the olfactory peduncle; 17.2 ± 10.9 for PBS mice and 154.6 ± 35.7 for fibril mice in the piriform cortex; 4.2 ± 2.2 for PBS mice and 140.8 ± 28.3 for fibril mice in the amygdala; 2.0 ± 0.9 for PBS mice and 102.6 ± 45.9 for fibril mice in the hippocampus; 27.4 ± 9.8 for PBS mice and 137.3 ± 50.7 for fibril mice in the entorhinal cortex; *p ≤ 0.05, **p ≤ 0.01, ***p ≤ 0.001 vs PBS, Student’s *t* test (*n* = 4–5 mice/group). **f** Polyclonal antibodies against pSer129 were preadsorbed with pSer129 blocking peptide or incubated alone for 24 h prior to application to tissue. Shown are adjacent sagittal sections from the same fibril-infused animal (1 h waterbath sonication), captured with equivalent exposures and intensity scaling. Images showing the overlap between monoclonal and polyclonal pSer129 immunostaining can be examined in Additional file 1: Figure S3. Additional preadsorption control data can also be viewed in Additional file 1: Figure S6

Mice infused in the right OB/AON with fibrils sonicated for 1 h in a waterbath displayed dense pSer129^+^ inclusions in the olfactory peduncle (defined here as the AON complex and the tenia tecta [27], Fig. 1, Additional file 1: Table S2). Mice infused with fibrils sonicated for 1 min with a probe exhibited far less α-synucleinopathy (Additional file 1: Table S2, Figure S1). PBS infusions did not result in any α-synucleinopathy. pSer129^+^ inclusions were formed in structures known to harbor anatomical connections with the OB/AON, such as the amygdala, the frontal, piriform, and entorhinal cortices, the nucleus of the lateral olfactory tract, the subiculum, and hippocampal CA1 [28–30] (Fig. 1a-c). Additional sparse pSer129 label was observed in some animals in CA2/CA3, the dentate gyrus, and the ectorhinal/perirhinal cortex (Additional file 1: Table S2; Fig. 1a-b), perhaps reflecting limited transynaptic transport to these sites from CA1, the subiculum, and the entorhinal cortex, all of which harbored dense inclusions (Fig. 1a-d) and project directly to the OB/AON [28, 31, 32]. We invite the reader to download Fig. 1 in high resolution and zoom in and out of the images in Fig. 1b as with a street map, in order to appreciate the full extent of the pSer129^+^ pathology and verify its anatomical sublocalization. Note that the image takes an extremely long time to fully populate the computer screen with high-resolution photos, not only upon opening the file, but also upon zooming in and navigating across brain regions. Most α-synuclein-containing inclusions were wrapped around Hoechst^+^ nuclei or found in processes (Fig. 1d). A blinded analysis revealed that the number of pSer129^+^ structures in the olfactory peduncle, the piriform and entorhinal cortices, amygdala (posteromedial cortical amygdala), and hippocampal formation (subiculum and CA1) were dramatically higher in fibril-injected mice (Fig. 1e). The SEM values in Fig. 1e reveal significant variability in hippocampal and entorhinal inclusion counts, perhaps because of slight differences in stereotaxic placements of infusate or inter-animal differences in vulnerability. The most robust pathology was evident in the AON, and this was far denser than in the OB itself (Additional file 1: Figure S1, S2, Fig. 1a, c*b*, e, Additional file 1: Table S2), consistent with reports that the AON is one of the main olfactory structures bearing Lewy pathology [3, 4, 15]. For AON subdivisions, please examine the higher magnification images provided beneath Additional file 1: Table S2 and the elegant work of Brunjes, Haberly, and colleagues [27–30]. pSer129 labeling was strikingly absent from the olfactory tubercle (Fig. 1a), which receives dense first-order projections from the OB/AON but does not send reciprocal projections back to olfactory structures [28–30], suggesting lack of anterograde transmission of α-synucleinopathy.

The development of dense perinuclear and neuritic inclusions following fibril infusions was confirmed with two independent (monoclonal and polyclonal) pSer129 antibodies (see polyclonal anti-pSer129 staining in Fig. 1f, Additional file 1: Figure S3). The staining with the polyclonal and monoclonal antibodies overlapped well when applied simultaneously (Additional file 1: Figure S3). For preadsorption controls for primary antibody specificity, we used the polyclonal pSer129 antibody because preadsorption controls with monoclonal antibodies are always expected to lead to loss of labeling regardless of which proteins they bind in tissue [33]. Preincubation of pSer129 antibodies with pSer129 blocking peptide led to dramatic loss of immunoreactivity relative to adjacent sections exposed to free, unbound primary antibodies (Fig. 1f). Omission of primary antibodies also led to loss of immunofluorescent signal (not shown).

**Fig. 2:**
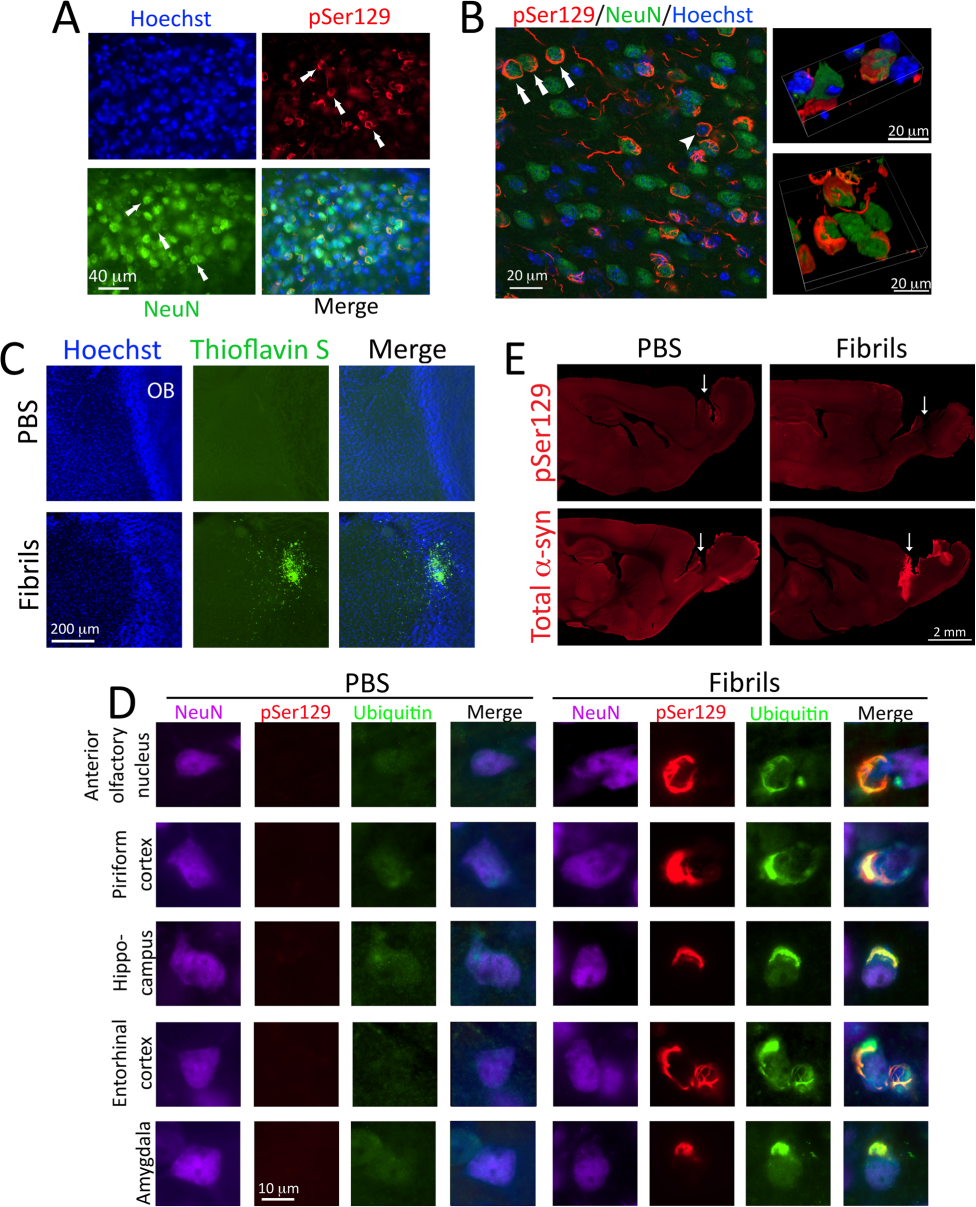
α-synucleinopathy is found within neuronal somata and some of it colocalizes with the ubiquitin marker of protein aggregates. **a** Two month-old mice were unilaterally infused with α-synuclein fibrils (5 μg) or an equal volume of phosphate-buffered saline (PBS) into the olfactory bulb and adjoining anterior olfactory nucleus (OB/AON). Fibrils were sonicated for 1 h in a waterbath prior to infusion. Three months later, sagittal brain sections were collected and stained with antibodies against pSer129 and the neuronal nuclear marker NeuN. Hoechst-labeled nuclei are shown in blue. Arrows point to some examples of the many triple-labeled cells in the AON. **b** Confocal microscopy showing the perinuclear localization of pSer129^+^ structures in the AON. Arrows: examples of Hoechst^+^/NeuN^+^/pSer129^+^ profiles. Arrowhead: Hoechst^+^/NeuN^−^/pSer129^+^ profile. For three-dimensional movies of pSer129^+^ and NeuN^+^ cells, see Additional file 2. **c** Sagittal sections through the OB/AON were stained with the Thioflavin amyloid stain. Nuclei were labeled blue with Hoechst. An additional sagittal level can be viewed in Additional file 1: Figure S4. **d** Colocalization of ubiquitin, pSer129, and NeuN in five different brain regions in mice that were sacrificed 3 months after fibril infusions in the OB/AON (1 h waterbath sonication). Some, but not all pSer129^+^ structures were ubiquitin^+^. The pSer129 and ubiquitin photos were captured from fibril and PBS-treated animals at the same exposures and intensity scaling. To view the results of the second ubiquitin antibody, please see Additional file 1: Figure S5. **e** Mice were infused in the OB/AON with 5 μg α-synuclein fibrils (1 h waterbath-sonicated) or PBS and perfused 1.5 h later. Brain sections were stained with rabbit antibodies against phosphorylated or total α-synuclein (red). The needle track is still apparent in the caudal OB at this early timepoint (white arrows) and reveals variability in the precise site of infusion, which would result in differences in inclusion counts in those afferent neurons terminating in the OB/AON in highly topographical manners. Images from PBS and fibril groups were captured at the same intensity scaling and exposure times. Differences in background staining are not the result of differential image processing. For a high-resolution version of this figure, please download the image at the link at the end of this article (Additional file 3) or email the corresponding author at leakr@duq.edu for access to the files

Next we characterized the nature of the inclusions formed three months following fibril infusions into the OB/AON. First, we confirmed that most perinuclear pSer129^+^ inclusions were formed within cells expressing the neuronal nuclear marker NeuN (Fig. 2a). This was further verified by a confocal analysis (Fig. 2b) and then by three-dimensional video analyses of the confocal images (see two videos in Additional file 2). The appearance of the somal α-synuclein inclusions—fibrous perinuclear strands—is similar to that reported in mouse tissue and some human Lewy bodies by Osterberg and colleagues [34]. Second, we examined whether the inclusions exhibited characteristics of Lewy pathology. The Thioflavin S stain for amyloid was applied to OB/AON tissue and densely labeled cellular structures at the infusion site but faintly or not at all elsewhere (Fig. 2c, Additional file 1: Figure S4). Ubiquitin staining, a traditional method for detecting Lewy-like pathology [22, 35], was present in some but not all pSer129^+^ structures, as shown with two antibodies against K48-linked ubiquitin (Fig. 2d, Additional file 1: Figure S5). These findings are consistent with the work of Osterberg et al. showing pSer129^+^ inclusions at progressive stages of maturity, with mature inclusions bearing hallmarks of Lewy pathology [34]. In addition, our ubiquitin staining pattern is consistent with the classic work of Spillantini et al. demonstrating that ubiquitin only stains a portion of all α-synuclein^+^ Lewy bodies and neurites [22].

**Fig. 3:**
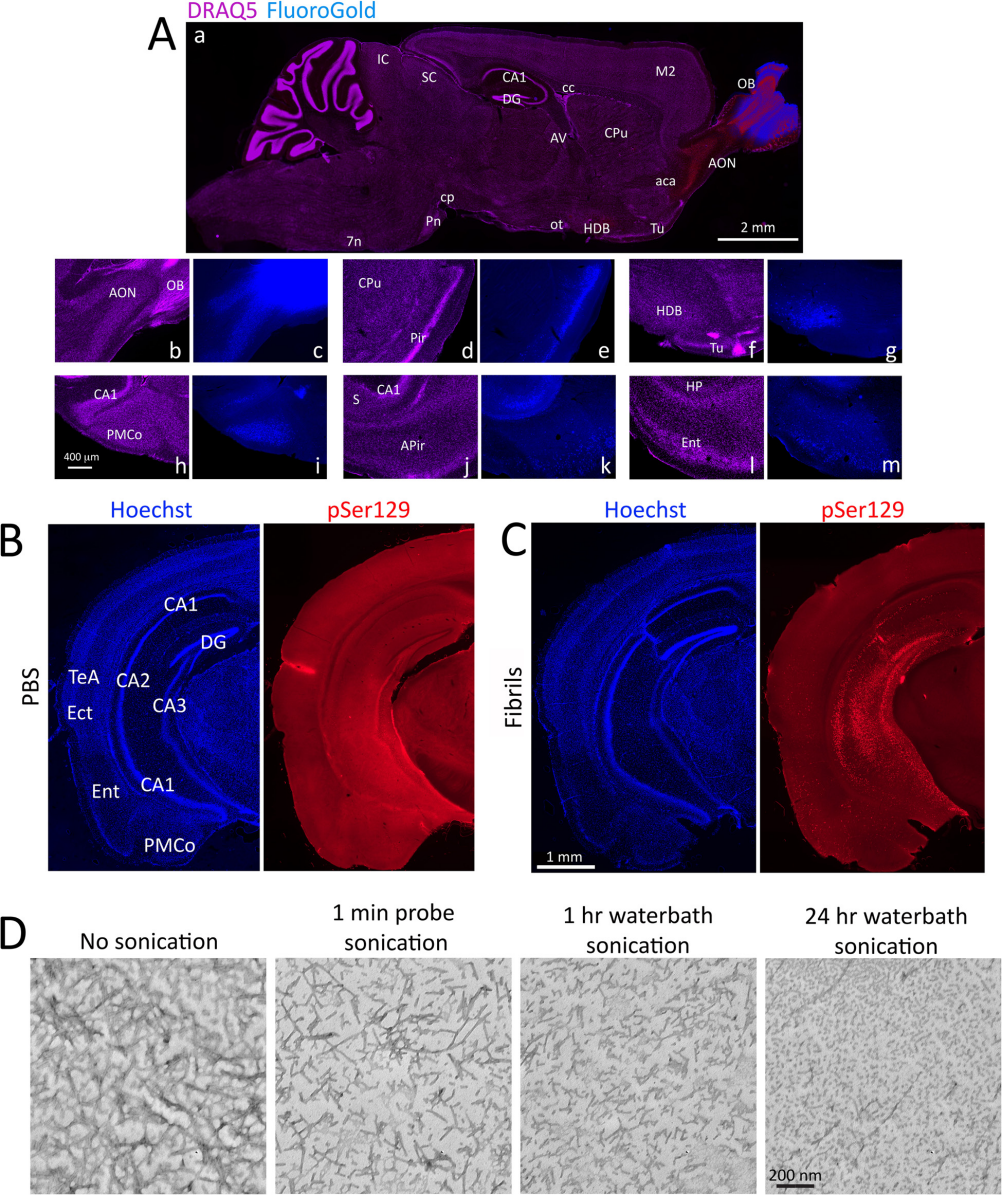
Areas harboring α-synucleinopathy send first-order projections to the site of infusion in the OB/AON. **a** The retrograde tracer FluoroGold (blue) was infused at the same stereotaxic coordinates as the α-synuclein fibrils. Animals were perfused after 7 days and sagittal sections were stained with the nuclear marker DRAQ5 (purple). Sections were imaged under UV and CY5 illumination. Dual-stained brain regions appear red (not purple or blue) in panel **a** *a*, such as the AON and the horizontal limb of the diagonal band (HDB). Dense FluoroGold label at multiple afferent sites is illustrated at higher magnification in panels *c*, *e*, *g*, *i*, *k*, and *m*. Nuclear DRAQ5 label on the same sections is shown in panels *b*, *d*, *f*, *h*, *j*, and *l*. Note the dense FluoroGold labeling in the horizontal limb of the diagonal band of Broca and the absence of FluoroGold label in the olfactory tubercle (Tu; *f-g*). Neither of these regions developed α-synucleinopathy. The olfactory tubercle is densely innervated by OB/AON projection neurons but does not project reciprocally back to the OB/AON. Abbreviations in Additional file 1: Table S1. **b-c** The transmission of α-synucleinopathy after infusions of waterbath-sonicated fibrils can be generalized to other neuroanatomical circuits. Two month-old mice were unilaterally infused with α-synuclein fibrils (5 μg) or an equal volume of phosphate-buffered saline (PBS) into CA2/CA3 of the hippocampus. Fibrils were sonicated for 1 h in a waterbath prior to infusion. Three months later, coronal brain sections were collected. Shown are stitched montages of pSer129 and Hoechst staining in the hippocampus and entorhinal cortex in PBS and fibril-infused animals—both groups were stained in parallel and captured at the same exposure and intensity scaling. The fibril injection in this animal extended from CA2/CA3 into CA1. Note the dense label in the entorhinal cortex, suggesting transmission of pathology through the perforant path, and the absence of label in the thalamus and most of the overlying cortex, demonstrating the lack of nonspecific uptake by neighboring sites from diffusion through the cerebrospinal fluid and interstitial space. For the antibody preadsorption control on coronal hippocampal tissue, see Additional file 1: Figure S6. To view the hippocampus at the same level after infusions of fibrils that were sonicated for 24 h, please examine Additional file 1: Figure S7. Abbreviations in Additional file 1: Table S1. **d** Electron micrographs of α-synuclein fibrils before and after sonication (1 min probe sonication and 1 h or 24 h waterbath sonication). Most sonicated fibrils were positively, not negatively stained with uranyl acetate. To view the pSer129 pathology in the entorhinal cortex and transentorhinal region in panel **c**, please zoom in and out of the high-resolution versions of these files at the link at the end of this article (Additional file 3) or email the corresponding author at leakr@duq.edu for access to the files

**Fig. 4:**
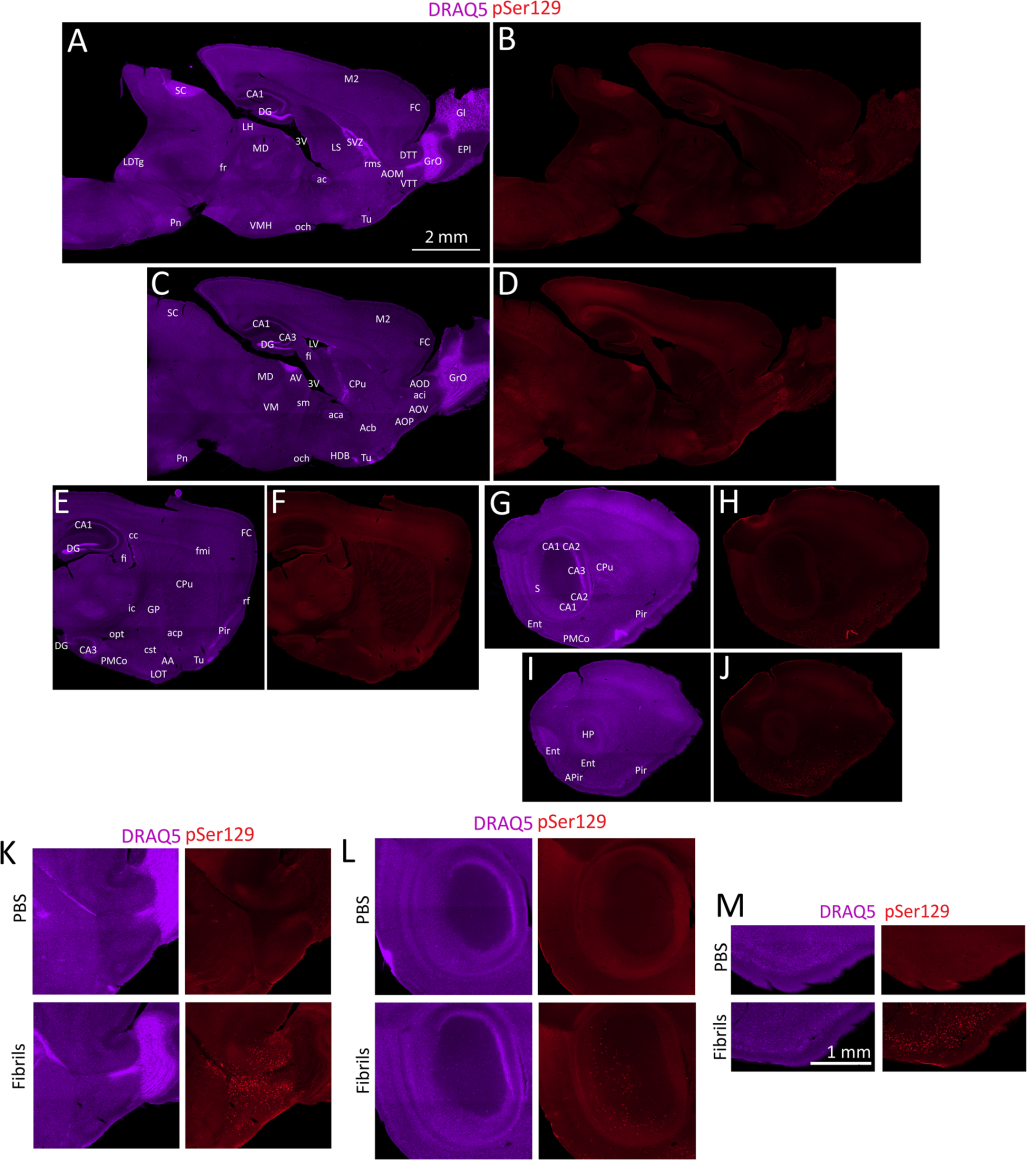
α-synucleinopathy transmission from olfactory structures in older mice. Mice (17 months old) were unilaterally infused in the OB/AON with α-synuclein fibrils (1 h waterbath sonication; see study on older animals in main text and in Additional file 1). Three months later, sagittal brain sections were collected and stained for DRAQ5 (**a**, **c**, **e**, **g**, **i**) and pSer129 (**b**, **d**, **f**, **h**, **j**). In order to fully appreciate the density of the inclusions and the neuroanatomical sublocalization, the reader must zoom in and out of the stitched pSer129 montages in the high-resolution versions of these files at the link at the end of this article (Additional file 3) or email the corresponding author at leakr@duq.edu for access to the files and examine panels **k**-**m**. The extent and density of the label in these fibril-infused animals should be contrasted with the lack of inclusions in large stitched montages of PBS-infused animals in Additional file 1: Figure S8. Abbreviations in Additional file 1: Table S1. **k**-**m** Higher magnification views of PBS versus fibril-infused cases. Shown are the olfactory peduncle (**k**), the hippocampus (**l**), and the piriform cortex (**m**). The entire brain section of these PBS cases can be fully examined for background staining in Additional file 1: Figure S8. The scale bar for **a-j** is in panel A and the scale bar for **k-m** is in panel **m**

Previous work has shown that the fibrils themselves are not phosphorylated [36]; the pSer129^+^ structures in the present study therefore likely demark endogenous, aggregated α-synuclein and not direct transport of injected material as in the Rey study [13]. This interpretation is consistent with the findings that 1) there was unexpectedly sparse pSer129 labeling in the OB itself—*the epicenter of the fibril infusions*—and 2) that there were no pSer129^+^ inclusions in animals sacrificed 1.5 h after fibril infusions (Fig. 2e). However, the latter animals exhibited extremely dense *total* (non-phosphorylated) α-synuclein immunoreactivity at the injection site relative to PBS-infused controls, effectively delineating the center of the infusion site in the caudal OB (Fig. 2e).

### Study 2: Retrograde tract-tracing of first-order afferent neurons that project to the site of infusion

The topography of the pSer129 label three months following fibril infusions into the OB/AON suggested that brain regions projecting directly to the site of infusion eventually developed inclusions. Thus, the retrograde tracer FluoroGold was infused into the OB/AON at the same stereotaxic coordinates in order to label first-order afferent neurons that project directly to the injection site (Fig. 3a). FluoroGold labeling of first-order projection neurons was evident at all inclusion-bearing sites, with the exception of the caudoputamen, accumbens, CA2/CA3, and dentate gyrus (Additional file 1: Table S2). Furthermore, dense and brightly stained FluoroGold^+^ neurons were additionally present in some areas not harboring any pSer129^+^ pathology, such as the horizontal limb of the diagonal band of Broca (Fig. 3a*a*, a*f-g*, Additional file 1: Table S2), which is known to project densely to the AON [28–30] but resisted the development of α-synucleinopathy (Fig. 1a, also see Fig. 4), suggestive of selective vulnerabilities. No retrogradely labeled FluoroGold^+^ cells were present in the olfactory tubercle (Fig. 3a*a*, a*f-g*, Additional file 1: Table S2), consistent with the lack of pSer129^+^ structures at this location (Fig. 1a, also see Fig. 4b, d, f) and with previous reports that the olfactory tubercle does not project to the OB/AON [28–30]. These findings suggest a lack of anterograde transmission of α-synucleinopathy, as argued above, or additional selective vulnerabilities.

### Study 3: Reproducibility of the fibril sonication protocol in a separate set of neuroanatomical circuits

In order to confirm that our sonication protocol was reproducible in a different set of neuroanatomical circuits, we examined the spread of α-synucleinopathy through the perforant path from CA2/CA3. These animals were infused with vehicle or fibrils sonicated for 1 h or 24 h in a waterbath, as 1 min-long probe sonication was less successful in Study 1. Animals were sacrificed three months later. Fibril infusions centered in CA2/CA3 of the hippocampus led to dense pSer129^+^ inclusions at the site of injection as well as CA1 and the dentate gyrus (Fig. 3b-c). The entorhinal cortex was densely labeled in fibril-infused animals, suggestive of successful α-synucleinopathy transmission through the perforant path, as in previous studies [24]. Additional pathology was evident in the ectorhinal/perirhinal region, which also projects into the hippocampal formation [37] and has been highlighted by Braak as an important node for the spread of Lewy pathology from the mesocortex into the neocortex in Braak stages 5 and 6 [3, 38]. Similar patterns were observed with the polyclonal pSer129 antibody, and this was again significantly reduced upon preadsorption (Additional file 1: Figure S6). The antibody control experiments—including colabeling with polyclonal and monoclonal antibodies—were performed on at least two separate occasions, on coronal hippocampal tissue from CA2/CA3 infusions and sagittal tissue from OB/AON infusions, and the results were in agreement.

Hippocampal pathology was denser in the 1 h waterbath-sonicated group relative to 24 h sonication (Additional file 1: Figure S7), possibly because of overheating and degradation of α-synuclein over the course of 24 h (the entire waterbath became hot). In order to visualize fibril morphology under different sonication conditions, we performed electron microscopy and observed reductions in fibril size with 1 min probe sonication, 1 h waterbath sonication, and 24 h waterbath sonication (Fig. 3d), as in previous work [39].

### Study 4: α-synucleinopathy transmission from the OB/AON of aged animals

In order to ensure that the transmission of α-synucleinopathy from the OB/AON into deeper rhinencephalic structures was reproducible, we injected a second set of animals in the OB/AON with PBS or fibrils. Based on the findings of the CA2/CA3 study (Study 3), the fibrils were sonicated for 1 h. As an additional control, the investigator performing the stereotaxic surgeries was blinded to the identity of the injected solutions. Animals were infused at 5 or 17 months of age and sacrificed three months later. The extent of pathology in older animals is illustrated in Fig. 4. The pSer129 label in these fibril-infused animals can be contrasted with the lack of inclusions in large stitched montages of PBS-infused animals in Additional file 1: Figure S8. These images from PBS mice can also be deeply examined for background staining with the pSer129 antibody. For the semi-quantitative analysis shown in Additional file 1: Table S2, all the two month-old animals from the first study were restained with pSer129 antibodies in parallel with all the older animals in order to control against inter-experimental variability in immunofluorescence. The relative degree of staining in each brain region was then rated by a blinded observer. Relative to young animals, some aged (20 month old) animals exhibited more inclusions in the ventral striatum (nucleus accumbens), which was not labeled retrogradely with FluoroGold and therefore does not project to the OB/AON injection site. These findings are consistent with prior reports, as Rey and colleagues also reported striatal and frontal cortical uptake of human α-synuclein following OB infusions [13]. Two additional areas had sparse and variable labeling in some animals and were not included in the table, such as the bed nucIeus of stria terminalis, which also projects to the OB/AON [40], and the brainstem immediately caudal to the ventral portion of the fasciculus retroflexus, in or near the parabrachial nucleus. Both of these regions also harbored some FluoroGold^+^ cells.

Three important caveats of our study must be conceded. First, rodents are macrosmatic animals (i.e., they depend heavily upon olfaction) and therefore may exhibit denser rhinencephalic interconnectivity than humans. It would therefore be premature to conclude that hippocampal Lewy pathology can be traced to the OB/AON in humans based only on rodent work. Second, recent studies suggest that labeling with the pSer129 antibody is subject to cross-reaction with neurofilament-L [39]. Indeed, we see abundant evidence of background staining, especially in fiber tracts (see edges of olfactory peduncle in Fig. 1c*a-b*, Additional file 1: Figure S1, photos below Additional file 1: Table S2, polyclonal pSer129 staining in Additional file 1: Figure S3a). To demonstrate that this background staining did not confound our interpretations, we have included enormous stitched montages of the whole brain in fibril and PBS groups (Figs. 1b and 4, Additional file 1: Figure S8). Although we cannot rule out all cross-reactivity with certainty [24], the background label is distinct from high-intensity labeling of inclusions, which is absent in PBS animals, as is evident from the stitched montages and the blinded quantification in Fig. 1e. It is also worth mentioning that the antibody specificity controls were successful (Fig. 1f, Additional file 1: Figure S3, S6). The third limitation of the present study is that our OB/AON model differs from the human condition in many important ways—for example, OB/AON infusions do not result in pathology in the olfactory tubercle or dense hippocampal pathology in CA2/CA3. In Parkinson’s disease, α-synucleinopathy is hypothesized to emerge first in enteric and olfactory structures and to spread from the OB/AON regions to the olfactory tubercle, piriform cortex, periamygdalear cortex, and entorhinal cortex (as observed in our study, with the exception of the olfactory tubercle), but “without advancing into non-cortical areas” [41]. Instead, most α-synucleinopathy is hypothesized to enter the human brain from the dorsal motor nucleus of the vagus [3, 38]. It is worth noting, however, that most of the sites exhibiting inclusions in Studies 1 and 4 are affected in Braak stages 1–4 of Parkinson’s disease [3, 38]. Despite the obvious differences between the models presented here and the human condition, the fibril model may be useful to test Heiko Braak’s hypotheses that neurons with long, thin, unmyelinated axons, and perhaps high metabolic rates, are more vulnerable to α-synucleinopathy than neurons with sturdy, myelinated axons [38, 42]. Such neurons may also exhibit impairments in proteasomal and autophagic degradation systems.

It is unlikely that diffusion and non-specific uptake of fibrils throughout all the interstitial space of the brain confounded our interpretations, as so many regions close to the OB/AON remained free of inclusions (e.g., Fig. 4) and the pSer129 label closely overlapped that of FluoroGold. Had non-specific uptake following fibril diffusion through the interstitial space been responsible for the pSer129 labeling, we would have expected a gradient of dense labeling close to the injection site and sparse labeling with increasing distance from the injection site, but this was not the case; for example, in the OB/AON studies there was dense label in distant locations such as the lateral and caudal entorhinal cortex but absence of label in closer sites such as the hypothalamus and thalamus (Studies 1 and 4). Similarly, in the CA2/CA3 study, the injection site in the hippocampus lies close to many areas that were free of pSer129 label (e.g., see thalamus and overlying cortex in Fig. 3c, Additional file 1: Figure S7). Instead, the inclusion topographies are most parsimoniously explained by preponderant transmission through anatomically linked circuits. Furthermore, the pSer129 antibodies are not simply labeling the injected fibrils following diffusion, because pSer129^+^ label was scarce at the OB infusion epicenter in all fibril-treated animals and the inclusions were absent 1.5 h following fibril injections, a timepoint at which there was a dramatic increase in total α-synuclein labeling in the OB/AON relative to PBS controls.

In conclusion, the present short report demonstrates that 1) α-synucleinopathy can spread from the rostrally and medially situated OB/AON all the way to the caudal recesses of the temporal lobe (hippocampal formation and entorhinal cortex) within three months, 2) the spread of pathology is consistent with retrograde transmission through neuroanatomical networks, although this obviously remains to be confirmed, 3) some first-order afferent sites retrogradely transport FluoroGold from the OB/AON but resist the development of α-synucleinopathy, and 4) age at fibril infusion does not make a robust difference in α-synucleinopathy transmission in the first three months. Although our findings are consistent with the olfactory vector hypothesis of Parkinson’s disease [2] and may be especially relevant to diffuse Lewy body disease or dementia with Lewy bodies, it is difficult to extrapolate from rodent data directly to humans. If such extrapolation is eventually validated, then our findings may have implications for neurological outcomes, such as those controlled by the piriform and entorhinal cortices, amygdala, hippocampus proper, and subiculum. For example, memory loss in Parkinson’s disease is associated with α-synuclein pathology in CA2, the amygdaloid complex, and the entorhinal cortex and with tissue atrophy in CA1, CA3, and the subiculum [43, 44]. Indeed, loss of olfaction is a harbinger of cognitive dysfunction and memory loss [45–47] and predicts mortality in senior citizens; 22 % to 40 % of older adults with anosmia are dead within 5 years, as opposed to 10 % of elderly subjects with intact olfaction [48, 49].

## Abbreviations

AON, anterior olfactory nucleus; OB, olfactory bulb; PBS, phosphate buffered saline; pSer129, phosphorylated alpha-synuclein (Serine 129)

## Additional files

Additional file 1: Materials and methods, tables, and supplemental figures S1-S8 (all supplemental figures are mentioned in the main text). (PDF 55506 kb) Additional file 2: Two mp4 movie files showing perinuclear localization of pSer129 signal (red) around NeuN^+^ nuclei (green). One movie shows a rotating cell and in the other video, the red pSer129 signal is peeled away to reveal the underlying green NeuN^+^ nucleus. (ZIP 1190 kb) Additional file 3: High resolution figures. (ZIP 277 mb)
