# Erratum to: Transmission of α-synucleinopathy from olfactory structures deep into the temporal lobe

**DOI:** 10.1186/s13024-016-0120-5

**Published:** 2016-07-27

**Authors:** Daniel M. Mason, Negin Nouraei, Deepti B. Pant, Kristin M. Miner, Daniel F. Hutchison, Kelvin C. Luk, John F. Stolz, Rehana K. Leak

**Affiliations:** 1 Division of Pharmaceutical Sciences, Duquesne University, 600 Forbes Ave, Pittsburgh, PA 15282 USA; 2 Department of Pathology, University of Pennsylvania, Philadelphia, PA 19147 USA; 3 Department of Biological Sciences, Duquesne University, Pittsburgh, PA 15282 USA

## Erratum

The errors and associated corrections described in this document concerning the original manuscript were accountable to the production department handling this manuscript, and thus are no fault of the authors of this paper. Additionally, the online manuscript has now been updated with these corrections accordingly.

- In Fig. 1c, the original article [1] showed Merge stains that did not correctly merge the adjacent Hoechst and pSer129 stains; these Merge stains should have shown transparent red (pSer129) stains for the purpose of overlapping the blue (Hoechst) stains.

**Fig. 1:**
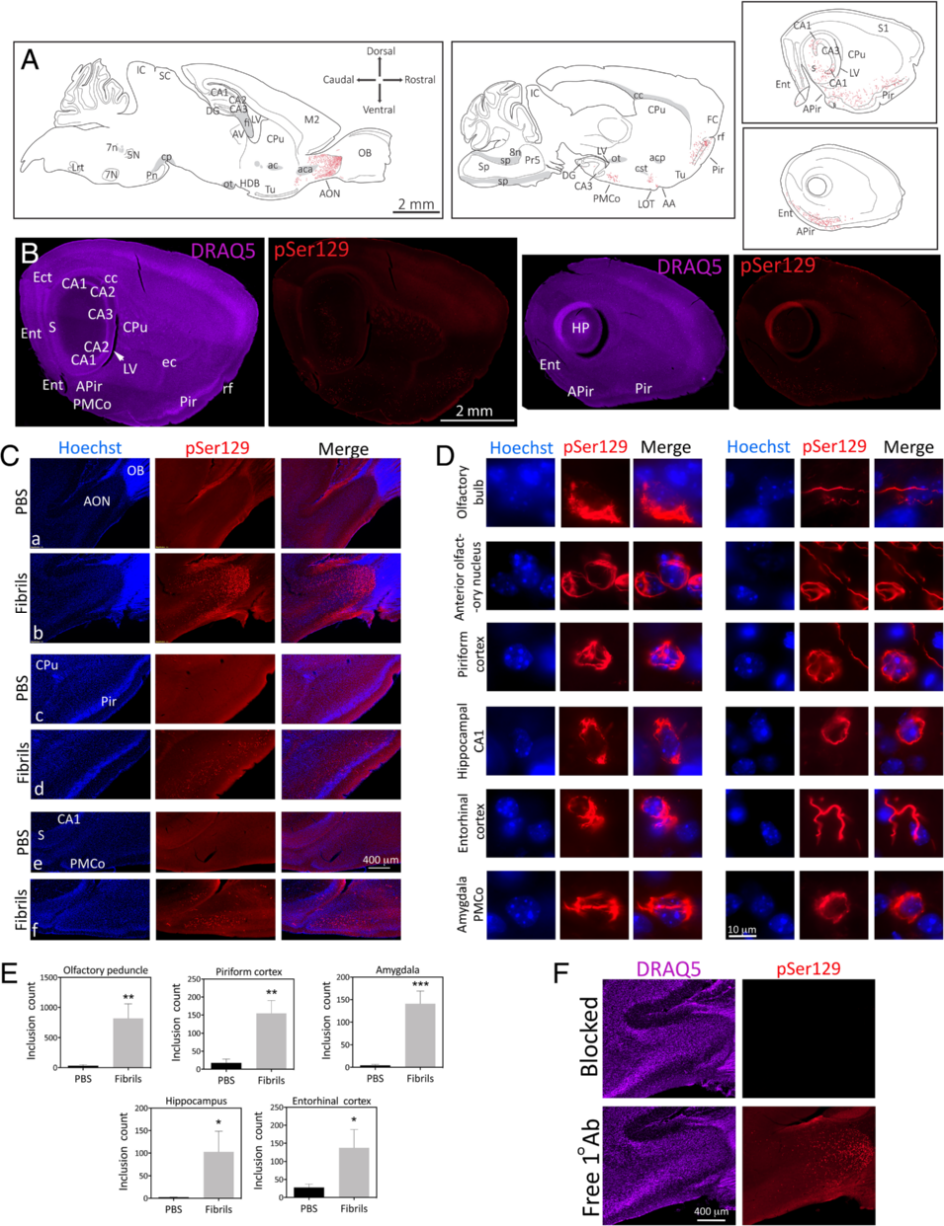
α-synucleinopathy is transmitted from the OB/AON to deeper rhinencephalic structures. Two month-old CD1 mice were unilaterally infused with α-synuclein fibrils (5 μg) or an equal volume of phosphate-buffered saline (PBS) into the olfactory bulb and adjoining anterior olfactory nucleus (OB/AON). Fibrils were sonicated for 1 h in a waterbath prior to infusion. Three months later, sagittal brain sections were collected and immunostained for pathologically phosphorylated α-synuclein (pSer129; red). Fibril and PBS groups were stained and photographed in parallel. **a** Large, high-quality microscopic photomontages of pSer129 and nuclear labeling were stitched together and viewed with Adobe Illustrator software on a tablet. Sagittal schematics of only obvious and clearly visible brain cytoarchitectonics (solid lines), myelinated fiber bundles (gray shading), pSer129^+^ neurites (red flourishes), and pSer129^+^ somal inclusions (red dots) were then traced with the pencil and paintbrush tools. All abbreviations are listed in Additional file 1: Table S1. **b** Examples of stitched photomontages of pSer129 immunostaining and DRAQ5 nuclear labeling following fibril infusions in the OB/AON (different sections than drawn in A). Please download the high-resolution supplemental versions of these files at the link at the end of this article (Additional file 3) or email the corresponding author at leakr@duq.edu for access to the files and zoom in and out of the stitched montages, in order to appreciate the density of the pathology, distinguish it from background, and judge the precise anatomical location. **c** pSer129 and nuclear Hoechst staining in the olfactory peduncle (*a-b*), the piriform cortex (*c-d*), and the hippocampus and amygdala (*e-f*) in PBS and fibril-infused animals. Abbreviations in Additional file 1: Table S1. **d** Images of pSer129^+^ inclusions and Hoechst-stained nuclei were captured using a 100× oil objective. The pSer129^+^ inclusions were perinuclear or found in processes (also see confocal images in Fig. 2b and Additional file 2, which shows two 3D-like videos of pSer129/NeuN labeled cells). The pSer129^+^ cell in the OB (uppermost row) was in the mitral cell layer, which is known to house large somata. Images of the sparse label in the OB can be viewed in Additional file 1: Figure S1, S2, and the images below Additional file 1: Table S2. **e** pSer129^+^ inclusion numbers were counted in ImageJ software by a blinded observer, after the same threshold values were applied across all images. Inclusion counts were 29.8 ± 20.2 (mean ± SEM) for PBS mice and 817.0 ± 242.5 for fibril mice in the olfactory peduncle; 17.2 ± 10.9 for PBS mice and 154.6 ± 35.7 for fibril mice in the piriform cortex; 4.2 ± 2.2 for PBS mice and 140.8 ± 28.3 for fibril mice in the amygdala; 2.0 ± 0.9 for PBS mice and 102.6 ± 45.9 for fibril mice in the hippocampus; 27.4 ± 9.8 for PBS mice and 137.3 ± 50.7 for fibril mice in the entorhinal cortex; *p ≤ 0.05, **p ≤ 0.01, ***p ≤ 0.001 vs PBS, Student’s *t* test (*n* = 4–5 mice/group). **f** Polyclonal antibodies against pSer129 were preadsorbed with pSer129 blocking peptide or incubated alone for 24 h prior to application to tissue. Shown are adjacent sagittal sections from the same fibril-infused animal (1 h waterbath sonication), captured with equivalent exposures and intensity scaling. Images showing the overlap between monoclonal and polyclonal pSer129 immunostaining can be examined in Additional file 1: Figure S3. Additional preadsorption control data can also be viewed in Additional file 1: Figure S6
- In the Fig. 1 legend, the article [1] should have had the additional line:
  “Please download the high-resolution supplemental versions of these files at the link at the end of this article (Additional file 3) or email the corresponding author at leakr@duq.edu for access to the files and zoom in and out of the stitched montages in order to appreciate the density of the pathology, distinguish it from background, and judge the precise anatomical location.”
- In the Fig. 2 legend, the article [1] should have had the additional line:
  “For a high-resolution version of this figure, please download the image at the link at the end of this article (Additional file 3) or email the corresponding author at leakr@duq.edu for access to the files.”
- In the Fig. 3 legend, the article [1] should have had the additional line:
  “To view the pSer129 pathology in the entorhinal cortex and transentorhinal region in panel c, please zoom in and out of the high-resolution versions of these files at the link at the end of this article (Additional file 3) or email the corresponding author at leakr@duq.edu for access to the files.”
- In the Fig. 4 legend, the article [1] should have had the additional line:
  “In order to fully appreciate the density of the inclusions and the neuroanatomical sublocalization, the reader must zoom in and out of the stitched pSer129 montages in the high-resolution versions of these files at the link at the end of this article (Additional file 3) or email the corresponding author at leakr@duq.edu for access to the files and examine panels k-m.”
