# Supplementary material for: Transmission of α-synucleinopathy from olfactory structures deep into the temporal lobe
Source: Mol Neurodegener. 2016 Jun 30;11:49. doi: 10.1186/s13024-016-0113-4 (PMC4929736; doi:10.1186/s13024-016-0113-4)
Supplement: Additional file 1: — Materials and methods, tables, and supplemental figures S1-S8 (all supplemental figures are mentioned in the main text). (PDF 55506 kb) [file 13024_2016_113_MOESM1_ESM.pdf]

Supplemental Information

# Transmission of α-synucleinopathy from olfactory structures deep into the temporal lobe

Daniel M Mason[1^], Negin Nouraei[1^], Deepti B Pant[1], Kristin M Miner[1], Daniel F Hutchison[1], Kelvin C Luk[2], John F Stolz[3], and Rehana K Leak*[1]

[1]Division of Pharmaceutical Sciences, Duquesne University, Pittsburgh PA 15282
[2]Department of Pathology, University of Pennsylvania, Philadelphia PA 19147
[3]Department of Biological Sciences, Duquesne University, Pittsburgh PA 15282

^ these authors did equivalent work

*** For correspondence:**

Rehana K. Leak, Ph.D.
Division of Pharmaceutical Sciences
Duquesne University
600 Forbes Ave
Pittsburgh PA 15282
TEL: 412.396.4734
FAX: 412.396.4660
leakr@duq.edu

# Materials and Methods

## 1. Fibril sonication

Recombinant wildtype mouse α-synuclein fibrils were generated as previously published (Luk et al., 2012a; Luk et al., 2012b; Luk et al., 2009; Volpicelli-Daley et al., 2014; Volpicelli-Daley et al., 2011). As fibril sonication parameters are extremely important in the transmission of pathology (Volpicelli-Daley et al., 2014), we used two different sonication techniques. First, we sonicated fibrils with a probe sonicator (Misonix model XL2020, Misonix Incorporated, Farmingdale NY) for sixty 0.5 sec pulses with a pause every 10 pulses to let the solution cool (~1 min total). The total volume sonicated with the probe was approximately 75 µL. For the alternative waterbath sonication, we sonicated approximately 10 µL of sample (5 mg/mL) in a 0.5 mL centrifuge tube, continuously for 1h or for 24h (Bransonic series model M1800, Branson Ultrasonics Corporation, Danbury CT). It is worth noting that this sonicator is inexpensive relative to what is commonly used for fibril sonication in the literature. Fibril stock solutions were frozen, but were *not* placed on ice during or after sonication. During waterbath sonication, Parafilm was thickly wrapped around the tube lid to prevent any entry of aerosolized water into the tube. Fibril sonication was always completed within approximately ~0.5-4 hours of direct infusion into the central nervous system. The remaining fibrils were discarded after the infusions were complete and no solutions were reused.

## 2. Animals and fibril infusions

Considerable efforts were made to minimize pain and suffering and the number of animals sacrificed was kept to the very minimum. All procedures were approved by the Duquesne University Institutional Animal Care and Use Committee (IACUC protocol 1403-03) and in compliance with the *NIH Guide*. Following IACUC approval, male CD1 mice from Charles River were bred in the Duquesne University animal facility. All animals were group-housed with free access to food and water in a 12:12 hour photoperiod. Mice were injected with one microliter of sterile phosphate-buffered saline (PBS) or 5 µg preformed α-synuclein fibrils of wild-type mouse origin, as described previously (Luk et al., 2012a). Unilateral injections were made in the OB/AON (AP +4.0, ML -1.0, DV -2.5 mm from top of the skull at bregma) or the hippocampus (AP -2.5, ML -2.8, DV -3.6 mm from top of the skull at bregma). All coordinates were first verified by injecting blue food dye into the targeted structure and ensuring accurate placement of the infusate. Unlike Rey and colleagues, we targeted the rostral AON in addition to the caudal OB because the AON is a major and obvious site of olfactory Lewy pathology in early Parkinson's disease (Beach et al., 2009; Braak et al., 2003; Daniel and Hawkes, 1992; Rey et al., 2013; Ubeda-Banon et al., 2010). Fibrils were infused over the course of 4 minutes (0.25 µL/min) followed by an additional 4-minute dwell period to facilitate diffusion into brain parenchyma instead of up the needle track during withdrawal. During surgeries, animals were fully anesthetized with 2% vaporized isoflurane. All animals were placed on a warm pad until recovery from anesthesia. For pain control, all animals were treated subcutaneously with 0.1 mg/kg buprenorphine immediately after surgery and topical lidocaine on the incision for three days thereafter.

*We injected fibrils or PBS in three separate studies to ensure the reproducibility of our observations (Study 1, 3, and 4)*. For the first study, 2.5 month-old male CD1 mice were infused with wildtype, mouse α-synuclein fibrils (5 µg; n=10) or an equal volume of PBS (1 µL; n=7) into the OB/AON. Six of the animals in the fibril group were injected with 1h waterbath-sonicated fibrils and 4 animals were injected with 1 min probe-sonicated fibrils. These mice were sacrificed 3 months following fibril infusions. In the second part of the first study, we infused PBS (n=4) or fibrils (n=4; 5 µg/1 µL, 1h waterbath-sonicated) into the OB/AON of two month-old animals and sacrificed the animals 1.5h later, as Rey and colleagues reported the most extensive spread of human α-synuclein protein from the mouse OB at this timepoint (Rey et al., 2013). For the third study, mice were infused in CA2/CA3 of the hippocampus proper with α-synuclein fibrils (n=8; 5 µg) or an equal volume of PBS (1 µL; n=4); for this experiment, the fibrils were sonicated in the waterbath for either 1h (n=4) or 24h (n=4). We did not target CA1 as in previous work (Luk et al., 2012a), because CA2/CA3 is a major site of hippocampal Lewy pathology in Parkinson's disease and diffuse Lewy body disease (Braak et al., 2003; Dickson et al., 1994; Flores-Cuadrado et al., 2016). In the fourth study, animals were infused in the OB/AON either with PBS (three 5 month-old mice and three 17 month-old mice) or 5 µg fibrils (in 1 µL; three 5 month-old mice and four 17 month-old mice) sonicated for 1h in a waterbath. In the fourth study, the experimenter performing the stereotaxic infusions was completely blinded to the identity of the infusate and the experimental group assignments. The mice in Study 3 and 4 were also sacrificed 3 months post-infusion.

**3. FluoroGold transport to afferent sites**

In Study 2, the OB/AON of five 2 month-old mice was infused with 300 nL of a 1.5% solution of the retrograde fluorescent tracer FluoroGold (FluoroChrome, LLC, Denver, CO) (Schmued and Fallon, 1986; Wessendorf, 1991) at the same coordinates as used in the fibril-injected animals (see Methods Section 2 above). Note that the sphere of diffusion of a tracer through the parenchyma of the brain is expected to be more dependent upon the chemical properties and concentration of the infusate than the initial volume of injection; the FluoroGold images inform us about the location of the center of the injection, not the effective size of the fibril injection *per se*. One week following stereotaxic infusions of FluoroGold, animals were perfused through the heart with saline and 4% formalin in 0.1 M phosphate buffer. Perfused brains were immersed in 30% sucrose in 10 mM PBS for at least 2d before sagittal brain sections were collected on a freezing microtome. Free-floating sections were stored in cryoprotectant (Watson et al., 1986) at -20 $^{o}$C until use. Cryoprotectant was washed off in PBS and sections were stained for 1h at room temperature with the infrared nuclear marker DRAQ5 (1:10,000 or 0.5 µM, Biostatus, Leicestershire, UK) diluted in 10 mM PBS with 0.3% Triton X-100, mounted onto Superfrost Plus slides (Fisher Scientific, Pittsburgh PA), dried, and coverslipped in FluoroMount G (Southern Biotech, Birmingham AL). Retrograde FluoroGold labeling and DRAQ5-stained nuclei were visualized under UV and CY5 illumination with 4× or 10× objectives (Olympus IX73, B&B Microscopes, Pittsburgh PA). Montages were stitched with CellSens software (Olympus).

**4. Histology and image analyses**

Three months following fibril or PBS infusions, mice were perfused as above and free-floating brain sections from both groups were immunostained in parallel. Prior to the immunohistochemical procedures, cryoprotectant was washed off in several exchanges of PBS and sections were heated to 80 $^{o}$C in 10 mM sodium citrate tribasic dihydrate (pH 8.5) for 30 min for antigen retrieval (Jiao et al., 1999). Sections were then cooled to room temperature, washed in PBS, and placed in a fish serum-based Odyssey block (LiCor, Lincoln NE) for 1h at room temperature followed by overnight incubation in primary antibodies at 4 $^{o}$C on a shaker. The blocking solution was composed of 50% Odyssey block in 10 mM PBS with 0.3% Triton X-100 and was also used for all antibody incubations. Following overnight incubation, unbound primary antibodies were rinsed off with three exchanges of PBS and sections were incubated in secondary antibodies for 1h at room temperature. All primary and secondary antibodies are listed in **Table S3**. Nuclei were stained with Hoechst and/or DRAQ5 reagents during the secondary antibody incubation period. Following three final washes in PBS, sections were mounted onto glass slides, dried, coverslipped, and viewed with epifluorescent microscopy. Images were captured using 4×, 10×, 40×, or 100× (oil) objectives. Grayscale images (12-bit depth) were pseudocolored for presentation.

For the inclusion count measurements on young animals (Study 1), the final n was 4-5 per group. One PBS-infused and 1 probe-sonicated fibril-infused animal died, 1 PBS animal was poorly perfused, and the OB of 1 animal injected with waterbath-sonicated fibrils was lost during processing. In the fourth study, one PBS-infused animal in the oldest group (17 months of age) died. Raw, grayscale TIFF images were opened in ImageJ (NIH, Bethesda MD) and a blinded observer used the threshold tool and the “analyze particles” function to count inclusion numbers in rhinencephalic structures in PBS and fibril-infused mice. Equivalent threshold values were always applied across all images during this analysis.

Schematized drawings were made with a Surface Tablet Pro (Microsoft, Redmond WA) on a large stitched microscopic image of α-synuclein, NeuN, or nuclear staining using freehand tools in Adobe Illustrator (Adobe Systems, San Jose CA), while consulting the Paxinos and Franklin atlas (Franklin and Paxinos, 2013) and zooming in and out frequently to ensure correct delineation of cytoarchitectonic boundaries and inclusion morphology. The style of the drawings was adapted from the Swanson brain atlas (Swanson, 2004).

**5. Thioflavin S staining**

The Thioflavin S staining protocol was adapted from work by Paumier and colleagues (Paumier et al., 2015). Sagittal brain sections of mice from the OB/AON study were affixed on glass slides and immersed in 10 mM PBS for 5 min, followed by immersion in 0.05% $KMnO_4$ / PBS for 20 min. Following two rinses in PBS (2 min each), sections were destained in 0.2% $K_2S_2O_5$ / 0.2% oxalic acid / PBS until the brown color dissipated (~1 min). Following five rinses in PBS (2 min each), sections were immersed in freshly filtered 0.0125% Thioflavin S / 40% EtOH / 60% PBS for 3 min in the dark. Finally, sections were differentiated in 50% EtOH / 50% PBS for 10 min, followed by four rinses in PBS (5 min each). Nuclei were stained with the Hoechst reagent (0.003 µM; Hoechst 33258, bisBenzimide) in 0.3% Triton X-

100 during the last PBS wash, followed by a final 5-min rinse in deionized water. Stained sections were dried, coverslipped, and viewed with epifluorescent microscopy as above.

**6. Controls for antibody specificity**

The monoclonal and polyclonal pSer129 antibodies led to the same inclusion staining patterns (see polyclonal antibody staining in **Fig.1F, Fig.S3, Fig.S6**). The background staining with these two antibodies, especially along fiber tracts, was also the same. We used the monoclonal antibody for the majority of our figures (unless indicated otherwise) because it can be used at a far lower dilution at less cost. For the preadsorption controls for primary antibody specificity, we used the polyclonal pSer129 antibody, because preadsorption controls with monoclonal antibodies are always expected to lead to less of signal regardless of which proteins the antibodies actually bind within tissue (Saper, 2005). For these control experiments, we incubated the polyclonal pSer129 antibody with 10-fold excess pSer129 blocking peptide (Cat.no. 188826, Abcam, Cambridge, MA) for 24h at 4 $^{o}$C prior to application to tissue. Adjacent sections from the same animal were exposed in parallel to free primary antibodies or blocked primary antibodies for 24h at 4 $^{o}$C, followed by secondary antibody incubations, washes, mounting, and microscopic imaging as described above. To control for secondary antibody specificity, primary antibodies were omitted from the immunohistochemical procedures and led to loss of fluorescent signal as expected.

**7. Confocal microscopy**

Sagittal sections from animals infused with fibrils in the OB/AON were stained with pSer129 and NeuN antibodies and the Hoechst reagent as above and imaged on a confocal microscope with a 40× oil objective using 405nm, 488nm, 561nm lasers (A1 Confocal/Nikon Eclipse TiE motorized inverted microscope; Melville, NY). Labeling was viewed with the NIS-Elements C acquisition and analysis software platform.

**8. Electron microscopy**

Fibrils—both untreated and sonicated—were visualized using whole-mount preparations with transmission electron microscopy. Samples were suspended on Formvar-coated 200 mesh copper grids (Electron Microscopy Sciences, Hatfield PA), and stained with 1% uranyl acetate. Samples were then observed on a JEM-1210 TEM at 60 kV (Jeol USA Inc., Peabody MA) and images were captured using an ORCA-HR digital camera (Hamamatsu, Middlesex NJ). Fibrils were both negatively and positively stained with uranyl acetate, with much more positive than negative staining in all the sonicated groups, as exemplified in the photomicrographs in **Figure 3D**.

**9. Statistical analyses**

Data are presented in figures as the mean + SEM. The unpaired Student's *t* test was employed for the inclusion count measurements. All measurements were blinded.

Table S1: Abbreviations

| | | | |
|---|---|---|---|
| 5N | motor trigeminal nucleus | ic | internal capsule |
| 7n | facial nerve | IC | inferior colliculus |
| 7N | facial nucleus | LH | lateral habenula |
| 8n | vestibulocochlear nerve | lo | lateral olfactory tract |
| AA | anterior amygdaloid area | LOT | nucleus of the lateral olfactory tract |
| ac | anterior commissure | Lrt | lateral reticular nucleus |
| aca | anterior commissure, anterior part | LSd | lateral septal nucleus, dorsal part |
| Acb | nucleus accumbens | LSv | lateral septal nucleus, ventral part |
| aci | anterior commissure, intrabulbar | LTDg | laterodorsal tegmental nucleus |
| acp | anterior commissure, posterior part | LV | lateral ventricle |
| AHi | amygdalohippocampal area | M2 | secondary motor cortex |
| AMG | amygdala | MD | mediodorsal thalamic nucleus |
| AOB | accessory olfactory bulb | MEA | medial amygdala |
| AOD | anterior olfactory area, dorsal | mlf | medial longitudinal fasciculus |
| AOE | anterior olfactory nucleus, external | OB | olfactory bulb |
| AOM | anterior olfactory nucleus, medial | och | optic chiasm |
| AON | anterior olfactory nucleus | ot | optic tract |
| AOP | anterior olfactory area, posterior | Pir | piriform cortex |
| AOV | anterior olfactory area, ventral | PMCo | posteromedial cortical amygdala |
| APir | amygdalopiriform transition area | Pn | pontine nuclei |
| AV | anteroventral thalamic nucleus | Pr5 | principal trigeminal nucleus |
| BLA | basolateral nucleus of amygdala | rf | rhinal fissure |
| BMA | basomedial nucleus of amygdala | rms | rostral migratory stream |
| CA1 | cornu ammonis, field 1 | S | subiculum |
| CA3 | cornu ammonis, field 3 | S1 | primary somatosensory cortex |
| cc | corpus callosum | SC | superior colliculus |
| CEA | central nucleus of amygdala | sm | stria medullaris |
| cp | cerebral peduncle | sp | spinal trigeminal tract |
| CPu | caudoputamen | Sp | spinal trigeminal nucleus |
| cst | commissural stria terminalis | SVZ | subventricular zone |
| DG | dentate gyrus | TeA | temporal association cortex |
| Ect | ectorhinal cortex | TT | tenia tecta |
| Ent | entorhinal cortex | Tu | olfactory tubercle |
| EPl | external plexiform layer, olfactory bulb | VDB | ventral diagonal band |
| FC | frontal cortex | VM | ventromedial thalamic nucleus |
| fi | fimbria | VMH | ventromedial hypothalamic nucleus |
| fmi | forceps minor of the corpus callosum | VTT | ventral tenia tecta |
| fr | fasciculus retroflexus | | |
| Gl | glomerular layer, olfactory bulb | | |
| GP | globus pallidus | | |
| GrO | granule cell layer of olfactory bulb | | |
| HDB | horizontal limb of the diagonal band | | |
| HP | hippocampus | | |

Table S2: Density of pSer129$^{+}$ or FluoroGold$^{+}$ label following α-synuclein fibril or Fluorogold infusions into the OB/AON

| | | α-synuclein fibrils | | | | | | | | | | | | | | | FluoroGold | | | | |
|---|---|---|---|---|---|---|---|---|---|---|---|---|---|---|---|---|---|---|---|---|---|
| | | Probe-sonicated | | | Waterbath-sonicated | | | | | | | | | | | | | | | | |
| | | 2 mo | 2 mo | 2 mo | 2 mo | 2 mo | 2 mo | 2 mo | 2 mo | 5 mo | 5 mo | 5 mo | 17 mo | 17 mo | 17 mo | 17 mo | 2 mo | 2 mo | 2 mo | 2 mo | 2 mo |
| **Region** | **Hemisph.** | 15-327 | 15-329 | 15-330 | 15-332 | 15-333 | 15-334 | 15-340 | 15-341 | 15-439 | 15-441 | 15-443 | 15-445 | 15-447 | 15-449 | 15-451 | 15-452 | 15-453 | 15-454 | 15-455 | 15-456 |
| Olfactory bulb | Ipsi (R) | • | • | • | • | • | •• | • | • | • | • | • | •• | •• | •• | • | ••••• | ••••• | ••••• | ••••• | ••••• |
| | Contra (L) | • | - | • | - | - | - | • | - | - | - | - | - | •• | •• | - | - | - | - | - | - |
| Anterior olfactory nucleus | Ipsi (R) | •• | • | • | •••• | •••• | ••••• | •• | ••• | ••• | ••••• | • | ••••• | •••• | ••••• | ••••• | ••••• | ••••• | ••••• | ••••• | ••••• |
| | Contra (L) | • | - | • | ••• | •••• | ••••• | •• | • | •• | ••• | • | ••••• | •• | •••• | ••• | ••••• | ••••• | ••••• | ••••• | ••••• |
| Frontal cortex | Ipsi (R) | - | - | - | • | •• | • | - | - | - | ••• | - | • | - | • | • | - | •••• | ••••• | •• | ••••• |
| | Contra (L) | - | - | - | - | - | • | • | - | - | • | - | • | - | - | - | - | •• | ••• | - | - |
| Accumbens | Ipsi (R) | - | - | - | - | - | - | - | - | - | • | - | ••• | • | ••• | • | - | - | - | - | - |
| | Contra (L) | - | - | - | - | - | - | - | - | - | - | - | • | - | • | - | - | - | - | - | - |
| Caudoputamen | Ipsi (R) | - | - | - | - | • | • | - | - | - | - | - | • | - | - | • | - | - | - | - | - |
| | Contra (L) | - | - | - | - | - | - | - | - | - | - | - | - | - | - | - | - | - | - | - | - |
| Horizontal limb of the diagonal band | Ipsi (R) | - | - | - | - | - | - | - | - | - | - | - | - | - | - | - | •• | ••• | ••• | ••• | ••• |
| | Contra (L) | - | - | - | - | - | - | - | - | | | - | - | - | - | - | - | - | - | - | - |
| Olfactory tubercle | Ipsi (R) | - | - | - | - | - | - | - | - | - | - | - | - | - | - | - | - | - | - | - | - |
| | Contra (L) | - | - | - | - | | - | - | - | - | - | - | - | - | - | - | - | - | - | - | - |
| Piriform cortex | Ipsi (R) | • | • | • | ••• | ••••• | ••••• | •••• | •• | ••• | •••• | •• | ••••• | ••• | ••••• | ••••• | •••• | •••• | •••• | •••• | ••••• |
| | Contra (L) | - | - | • | • | •• | ••• | • | • | • | ••• | - | ••• | • | •• | • | •• | •• | ••• | •• | ••• |
| Nucleus of the lateral olfactory tract | Ipsi (R) | - | - | – | • | ••• | •••• | •• | •• | • | ••• | • | ••• | •• | •••• | •• | •••• | ••• | ••• | •• | ••• |
| | Contra (L) | - | - | - | • | • | ••• | • | - | - | • | - | • | - | • | ••• | ••• | •• | •• | - | •• |
| Cortical amygdaloid nuclei | Ipsi (R) | - | - | – | •• | •••• | •••• | ••• | •• | • | ••••• | - | •••• | ••• | •••• | •••• | •••• | ••• | •••• | ••• | •••• |
| | Contra (L) | - | - | - | - | •• | • | - | - | - | • | - | •• | - | - | •• | •• | • | •• | • | •• |
| Amygdalo-piriform transition area | Ipsi (R) | • | - | - | •••• | •••• | ••• | •••• | •• | • | •• | - | •••• | ••• | ••• | •••• | ••• | •• | ••• | • | ••• |
| | Contra (L) | - | - | - | • | •• | • | • | - | - | • | • | •• | - | • | • | • | - | - | - | - |
| Hippocampus (CA1) | Ipsi (R) | - | - | - | •• | •••• | ••• | •• | • | • | •••• | - | ••• | • | •• | ••• | •• | •• | ••• | •• | ••• |
| | Contra (L) | - | - | - | - | • | - | - | - | - | • | - | • | - | - | • | - | - | - | - | - |
| Hippocampus (CA2/CA3) | Ipsi (R) | - | - | - | • | ••• | •••• | • | - | • | ••• | - | •• | - | • | • | - | - | - | - | - |
| | Contra (L) | - | - | - | • | • | - | • | - | - | - | - | • | - | • | - | - | - | - | - | - |

| | | | | | | | | | | | | | | | | | | | | | |
|---|---|---|---|---|---|---|---|---|---|---|---|---|---|---|---|---|---|---|---|---|---|
| Hippocampus (dentate gyrus) | Ipsi (R) | - | - | - | • | • | • | • | • | - | • | - | •• | - | •• | • | - | - | - | - | - |
| | Contra (L) | - | - | - | • | • | • | - | - | - | | - | • | - | - | - | - | - | - | - | - |
| Subiculum | Ipsi (R) | - | - | - | • | •••• | ••• | •• | • | • | •••• | - | ••• | • | •• | •• | • | • | •• | - | •• |
| | Contra (L) | - | - | - | • | • | - | - | - | - | • | - | •• | - | - | • | - | - | - | - | - |
| Entorhinal cortex | Ipsi (R) | • | - | - | ••••• | ••••• | ••• | ••• | •• | • | •••• | - | ••••• | ••• | •••• | •••• | • | • | ••• | •• | •• |
| | Contra (L) | - | - | - | •• | •• | - | • | - | • | •• | - | ••• | - | • | • | - | - | • | - | • |

CD1 mice were infused in the OB/AON with recombinant, wildtype mouse α-synuclein fibrils sonicated for 1 min with a probe or 1h in a waterbath. Sagittal brain sections were stained with pSer129 antibodies 3 mo later. Separate mice were infused with FluoroGold in the OB/AON and sagittal brain sections were examined for retrogradely labeled cells 7d later. Animal IDs are shown in the fourth row. pSer129 or FluoroGold labeling is semi-quantitatively depicted in select regions in the ipsilateral (red) or contralateral (black) hemispheres from • to •••••(from sparsest to densest) for individual mice (see **Fig.1** for statistical analyses of inclusion counts). Absence of label is depicted by "-." For examples of sparsest •(**one dot**) to densest label •••••(**five dots**) see images directly below this table. The listed age of the animal is the age at infusion. All animals were sacrificed 3 mo following surgery (5, 8, 20 months). This table was generated by a blinded observer, with the exception of the label for the probe-sonicated and FluoroGold-infused animals, where it was obvious which group they belonged to. The variability in this table is typical of tract-tracing studies, and is likely explained by small variations in needle placement and differences in the sphere of diffusion of the infusate. Alternatively, there might be inter-individual variability in vulnerability to α-synucleinopathy. Abbreviations **in Table S1**

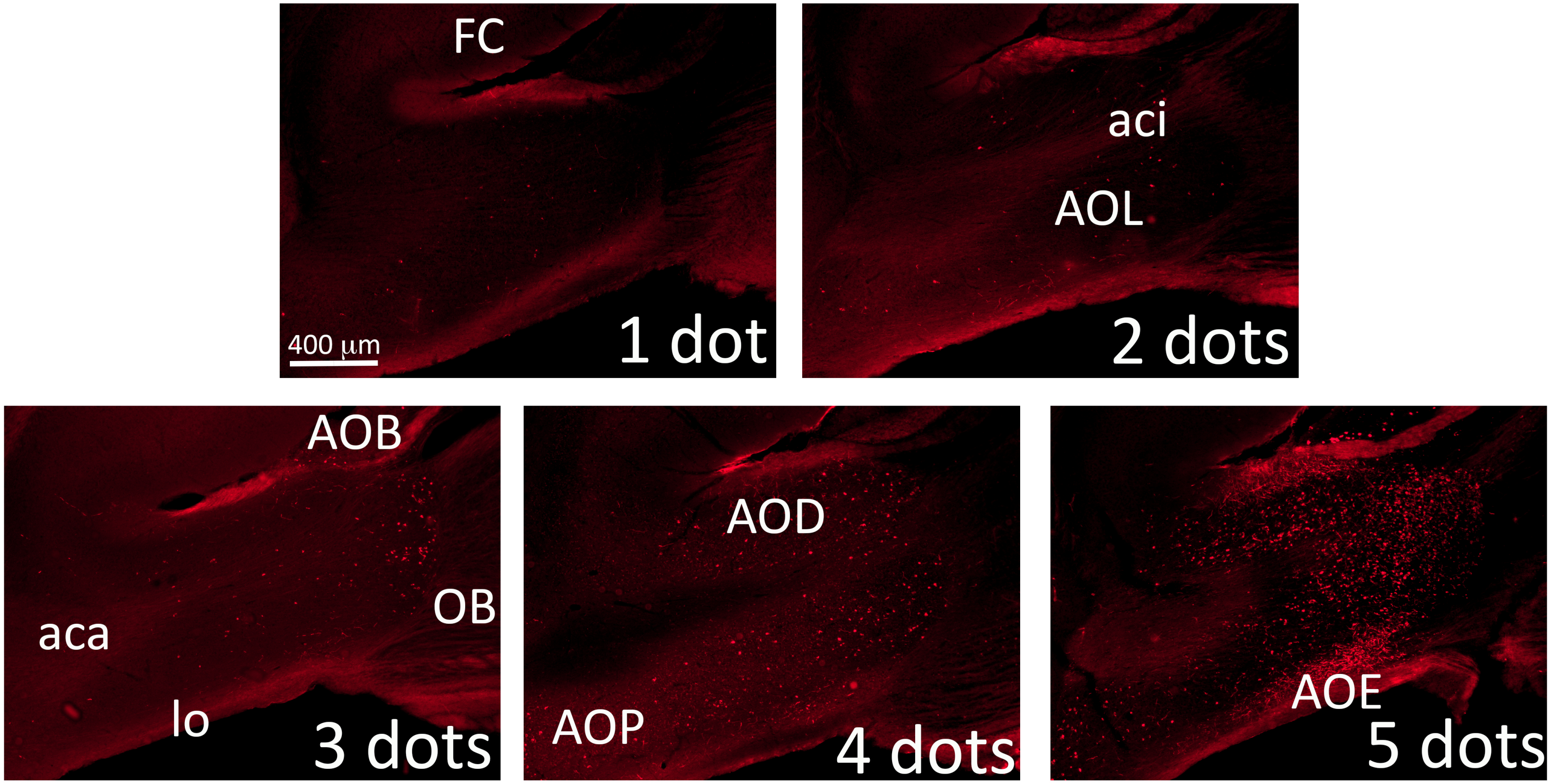

Table S3: Antibodies

| Primary Antibody | Source | Company | Catalog # | Lot # | Dilution |
|---|---|---|---|---|---|
| Anti-α-synuclein (pSer129) (aa 124 – 134; AYEMPS$^{P}$EEGYQ ) | Mouse | Gift from Kelvin Luk (81A) (Waxman and Giasson, 2008) | - | - | 1:5000 |
| Anti-α-synuclein (pSer129) (aa 127-131; MPS$^{P}$EE) | Rabbit | Abcam | Ab59264 | GR52476-25 | 1:300 |
| Anti-total-α-synuclein (aa 1-140) | Rabbit | Abcam | Ab155038 | GR117815-9 | 1:500 |
| Anti K48-linked ubiquitin | Rabbit | Millipore | 05-1307 | 2299608 | 1:500 |
| Anti K48-linked ubiquitin | Rabbit | Cell Signaling Technology | 4289S | 1 | 1:500 |
| Anti-NeuN | Guinea pig | Millipore | ABN90 | 2031353 | 1:6000 |

| Secondary Antibody | Source | Company | Catalog # | Lot # | Dilution |
|---|---|---|---|---|---|
| Anti-guinea pig @ 790 | Donkey | Jackson ImmunoResearch | 706-655-148 | 106036 | 1:1000 |
| Anti-guinea pig@ 647 | Donkey | Jackson ImmunoResearch | 706-605-148 | 123960 | 1:700 |
| Anti-guinea pig @ 488 | Donkey | Jackson ImmunoResearch | 706-545-148 | 108077 | 1:1000 |
| Anti-mouse @680 | Donkey | Jackson ImmunoResearch | 715-625-151 | 106244 | 1:1000 |
| Anti-mouse @ 555 | Goat | Invitrogen | A21424 | 1141876 | 1:1000 |
| Anti-mouse @ 488 | Donkey | Life Technologies | A21202 | 1423052 | 1:800 |
| Anti-rabbit @ 647 | Donkey | Jackson ImmunoResearch | 711-605-152 | 123104 | 1:1000 |
| Anti-rabbit @ 555 | Goat | Life Technologies | A21429 | 1562309 | 1:800 |
| Anti-rabbit @ 488 | Donkey | Jackson ImmunoResearch | 711-545-152 | 120705 | 1:700 |
| Anti-rabbit@ 546 | Goat | Life Technologies | A11035 | 1579044 | 1:500 |
| Anti-mouse @ 488 | Goat | Life Technologies | A11029 | 1550911 | 1:500 |

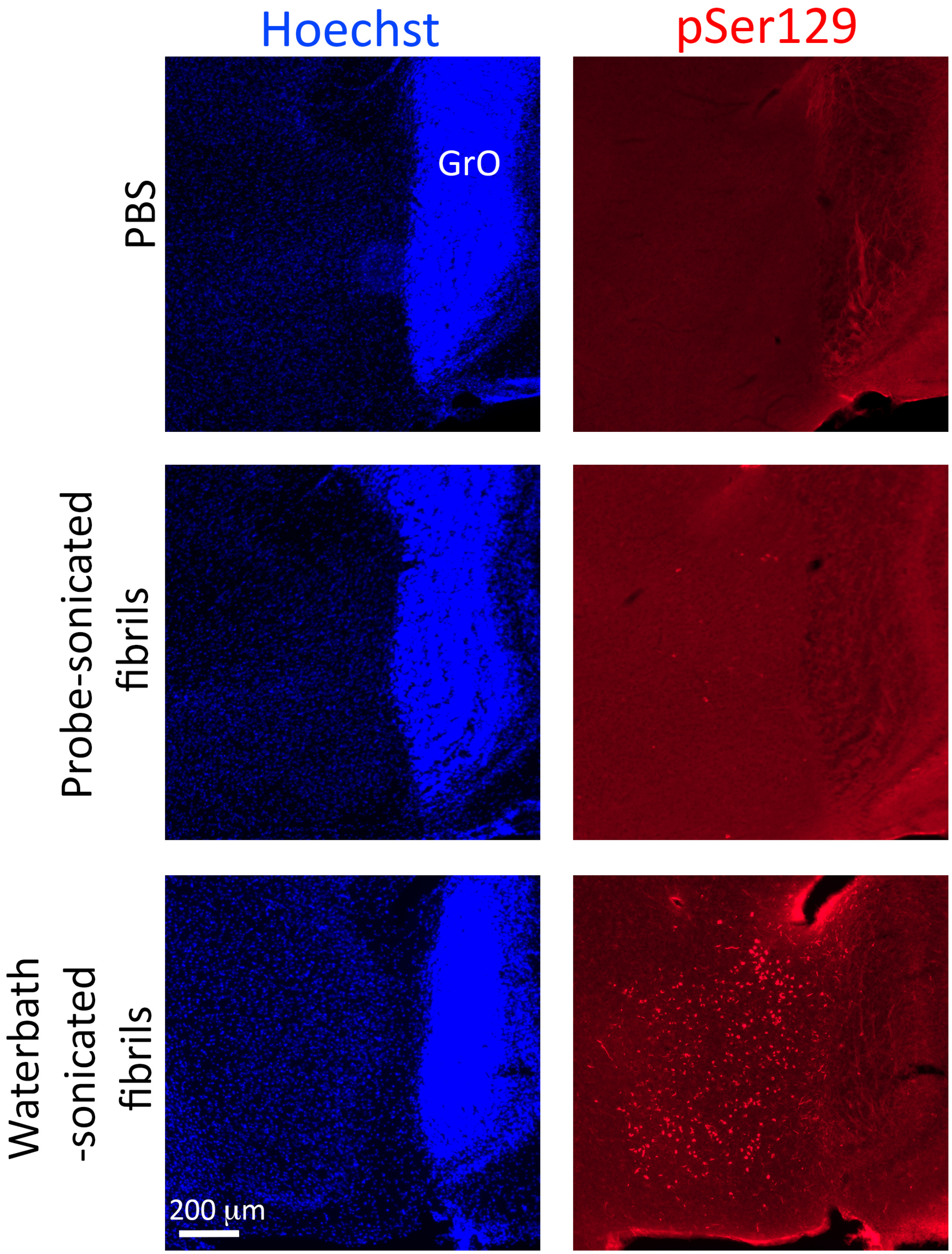


**Fig.S1** Mice were infused in the OB/AON with PBS or 5 μg α-synuclein fibrils (1 min probe-sonicated or 1h waterbath-sonicated). Sagittal brain sections were collected three months later and labeled with pSer129 antibodies (red) and the Hoechst nuclear stain (blue). The densest pSer129 label was in the AON and tenia tecta of the waterbath-sonicated animals (shown above). Only few inclusions were present in the animals injected with probe-sonicated fibrils. GrO, granule cell layer of the olfactory bulb

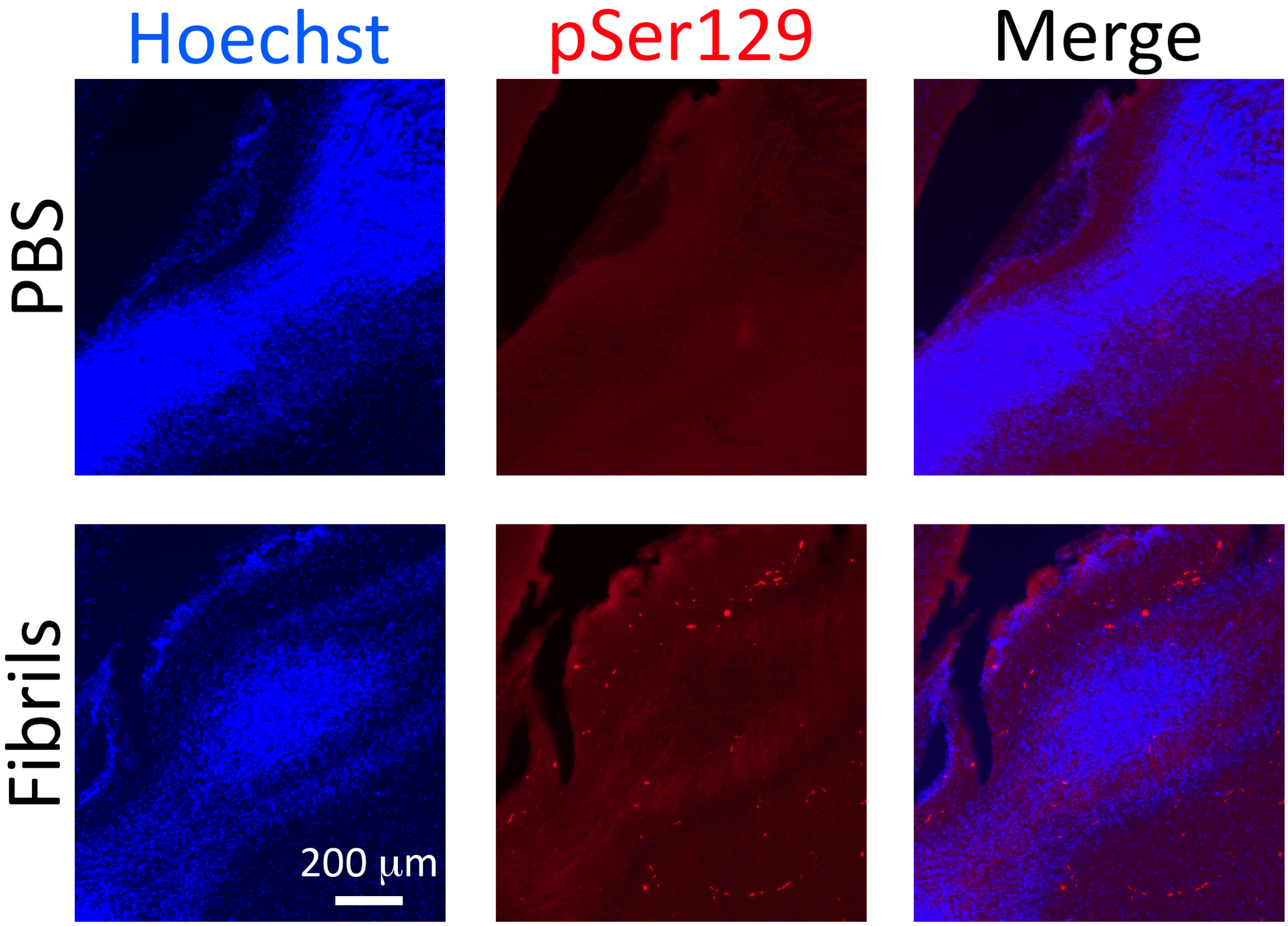


**Fig.S2** Mice were infused in the OB/AON with PBS or 5 μg α-synuclein fibrils (1h waterbath-sonicated). Sagittal brain sections were collected three months later and labeled with pSer129 antibodies (red) and the Hoechst nuclear stain (blue). The granule cell layer of the dorsal OB is shown. Only few inclusions were present in the OB in fibril-infused animals

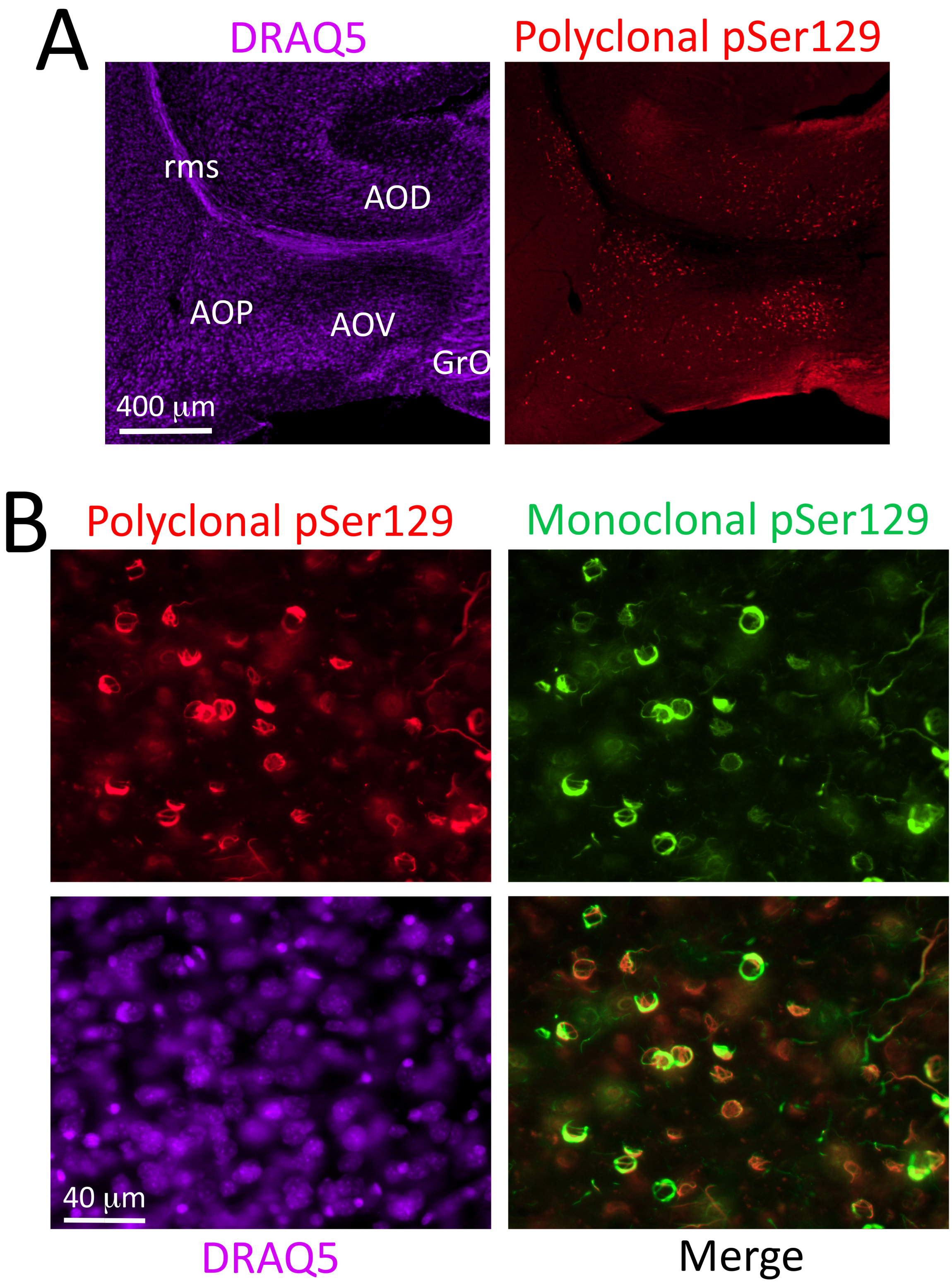


**Fig.S3** Mice were infused in the OB/AON with PBS or 5 μg α-synuclein fibrils (1h waterbath-sonicated). Sagittal brain sections were collected three months later. **(A)** Low power view of dense inclusions in the OB/AON, labeled with the polyclonal pSer129 antibodies (red). The DRAQ5 nuclear stain (purple) was applied to reveal cytoarchitectonic boundaries. **(B)** Polyclonal pSer129 antibodies (red) and monoclonal pSer129 antibodies (green) were applied simultaneously and visualized with distinct secondary antibodies. The merged image does not include the DRAQ5 staining because it overwhelmed the other labeling. A limited number of immunoreactive structures exhibited more staining with one antibody or the other, perhaps reflecting heterogeneity of the antibody clones, exposure of distinct epitopes within misfolded, aggregated protein clumps, or competition between antibodies binding the same epitope. For the sequence of the immunogens, please consult **Table S3**.

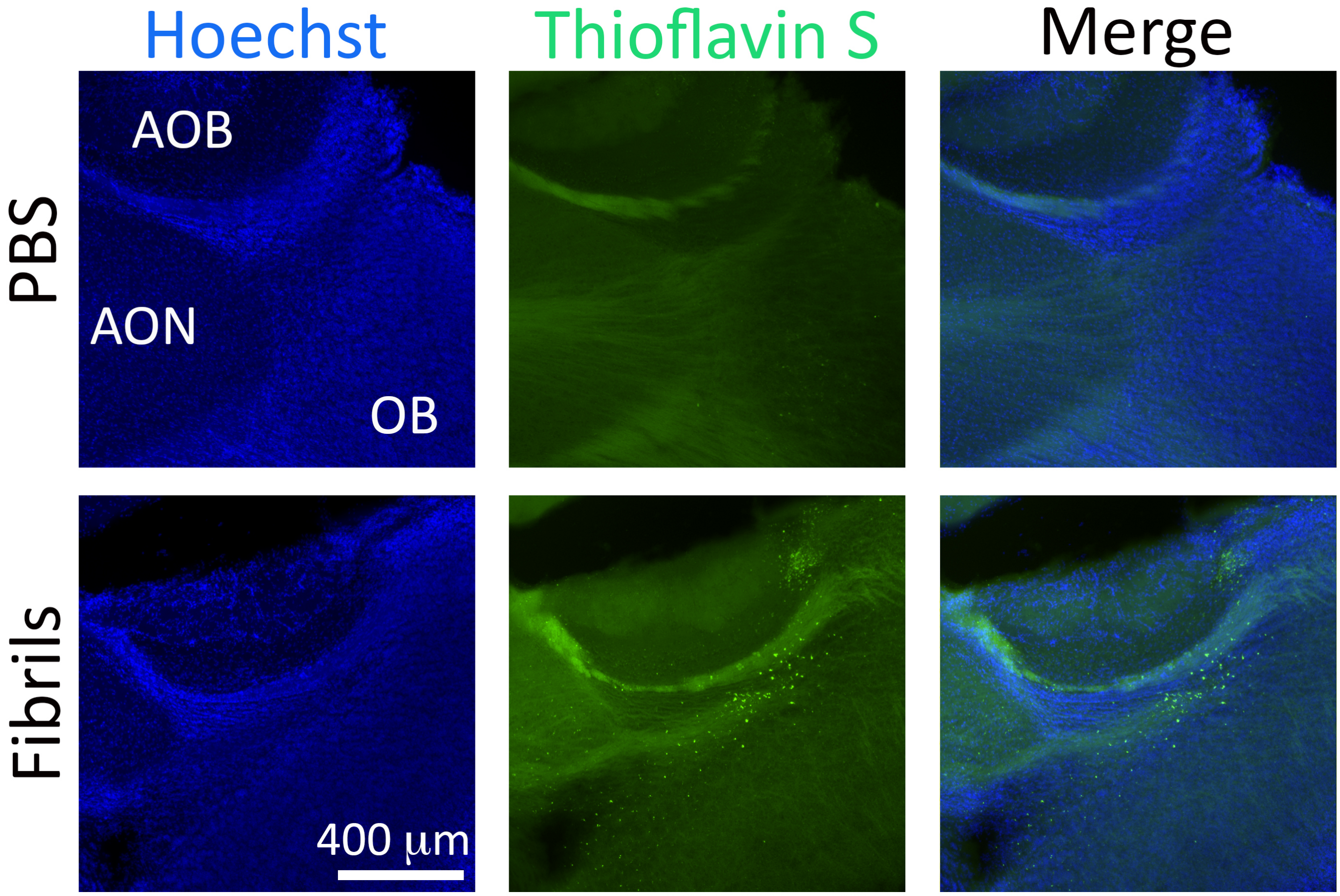


**Fig.S4** Mice were infused in the OB/AON with PBS or α-synuclein fibrils (1h waterbath-sonicated). Sagittal brain sections were collected three months later and labeled with the Thioflavin S stain for amyloid (green) and the Hoechst nuclear stain (blue). Amyloid staining was apparent at the site of fibril injection in the dorsal OB/AON only. AOB, accessory olfactory bulb

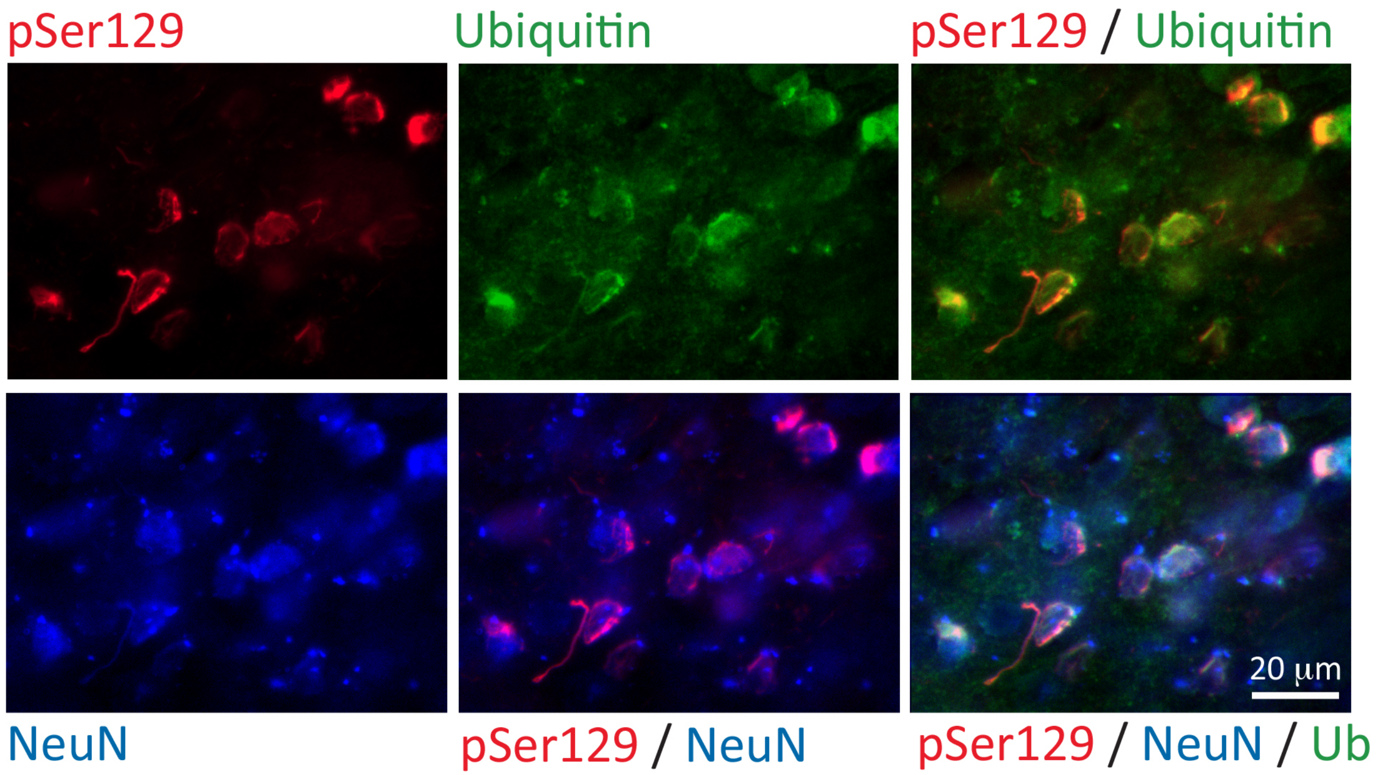


**Fig.S5** Mice were infused in the hippocampus with α-synuclein fibrils (1h waterbath-sonicated). Coronal brain sections were collected three months later and labeled for K48-linked polyubiquitin (green), pSer129 (red), and the neuronal nuclear marker NeuN (blue). Some but not all pSer129$^{+}$ structures in the amygdala (shown) contained dense K48-linked polyubiquitin. This figure shows the staining with the Cell Signaling Technologies anti-ubiquitin antibody. **Figure 2D** in the main text shows much more robust staining with the Millipore anti-ubiquitin antibody. See **Table S3** for antibody data

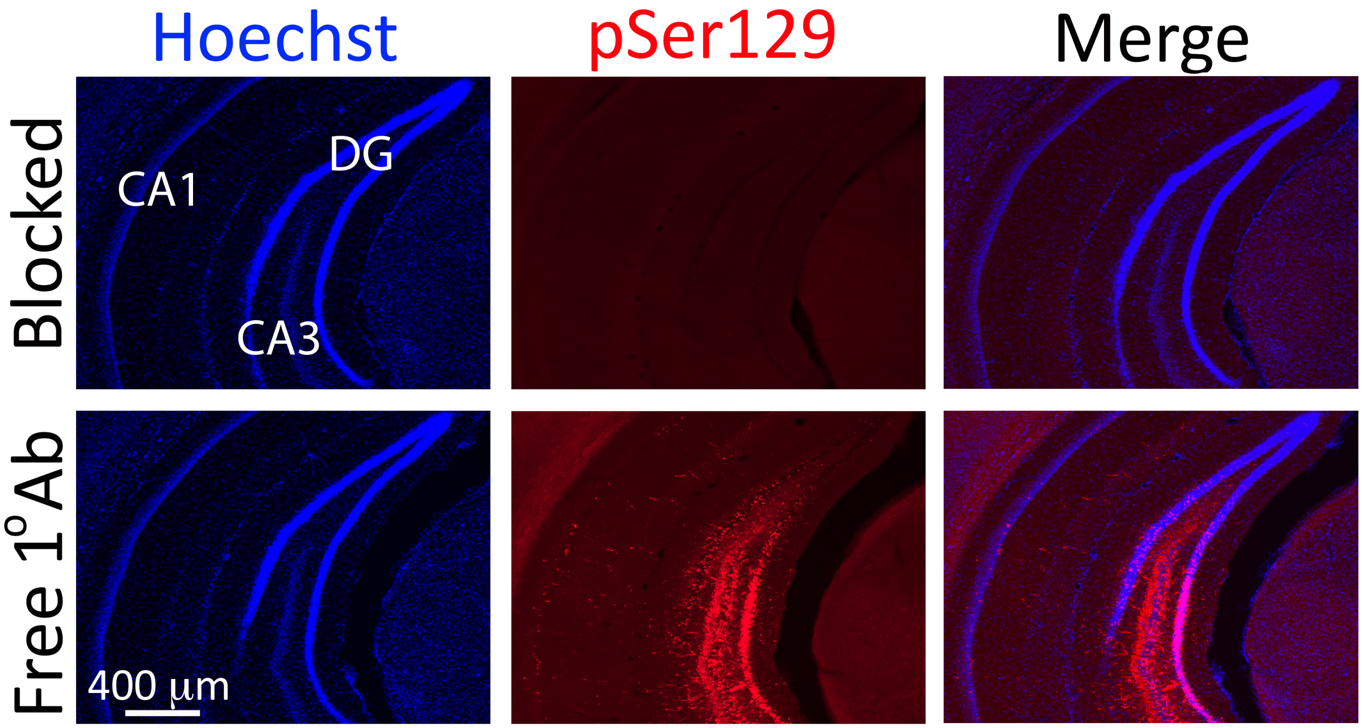


**Fig.S6** Mice were infused in the hippocampus with 5 µg α-synuclein fibrils (1h waterbath-sonicated). Coronal brain sections were collected three months later. Adjacent brain sections from the same animal were exposed either to free, unbound pSer129 polyclonal antibodies or preadsorbed pSer129 polyclonal antibodies. Preadsorption reduced the staining of inclusions (top panels). For the preadsorption control on OB/AON tissue, please examine **Fig.1F**. CA2/CA3, cornu or corpus ammonis; DG, dentate gyrus

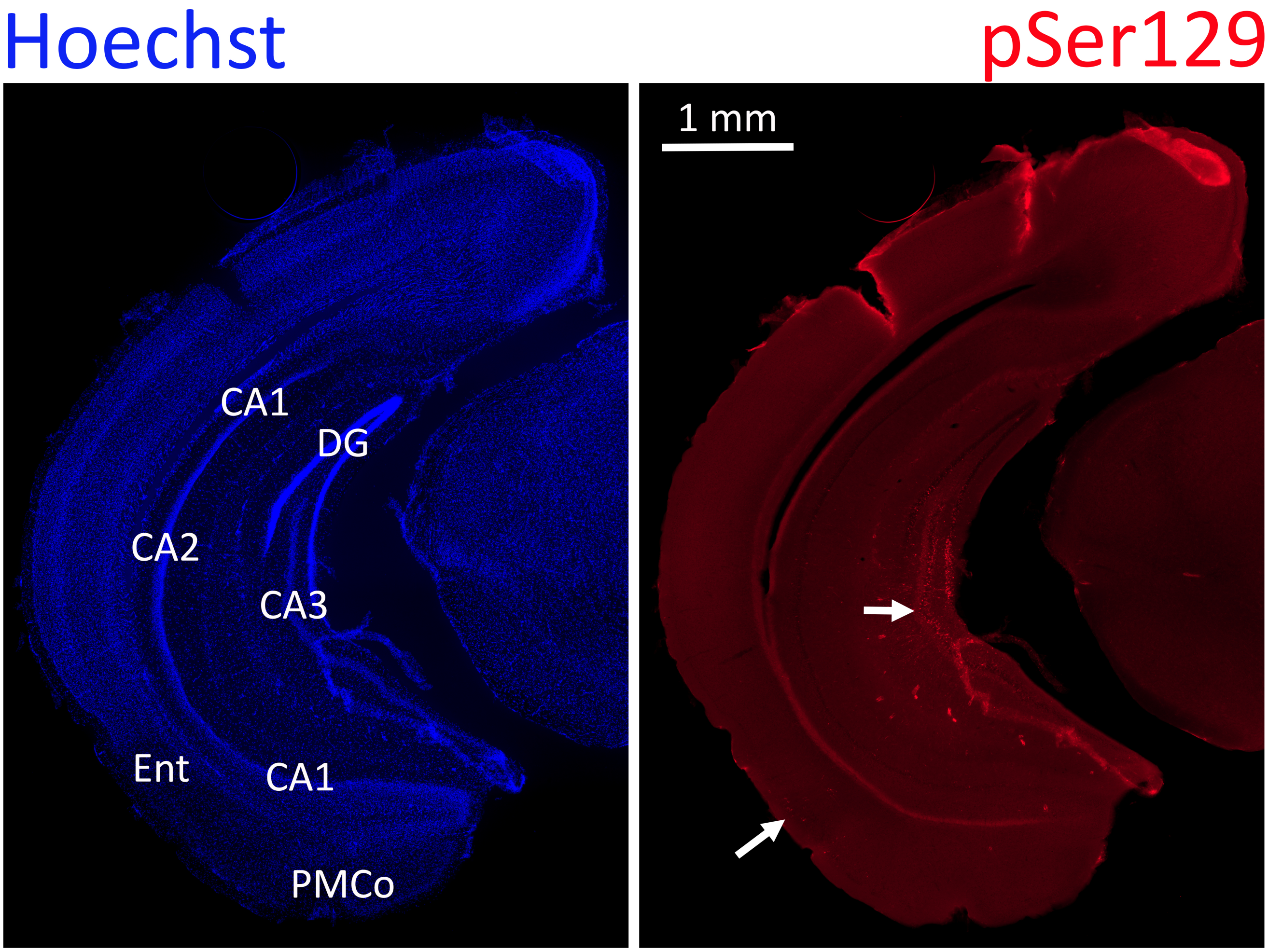


**Fig.S7** Mice were infused with α-synuclein fibrils (24h waterbath-sonicated) into CA2/CA3 of the hippocampus and sacrificed 3 months later. Coronal brain sections were stained with Hoechst (blue) and antibodies against pSer129 (red). The most inclusions were observed in the dentate gyrus (DG) and CA3 (see white horizontal arrow). Sparse inclusions were also observed in the entorhinal cortex (Ent; white diagonal arrow). Please zoom into the figure to distinguish the inclusions from background label

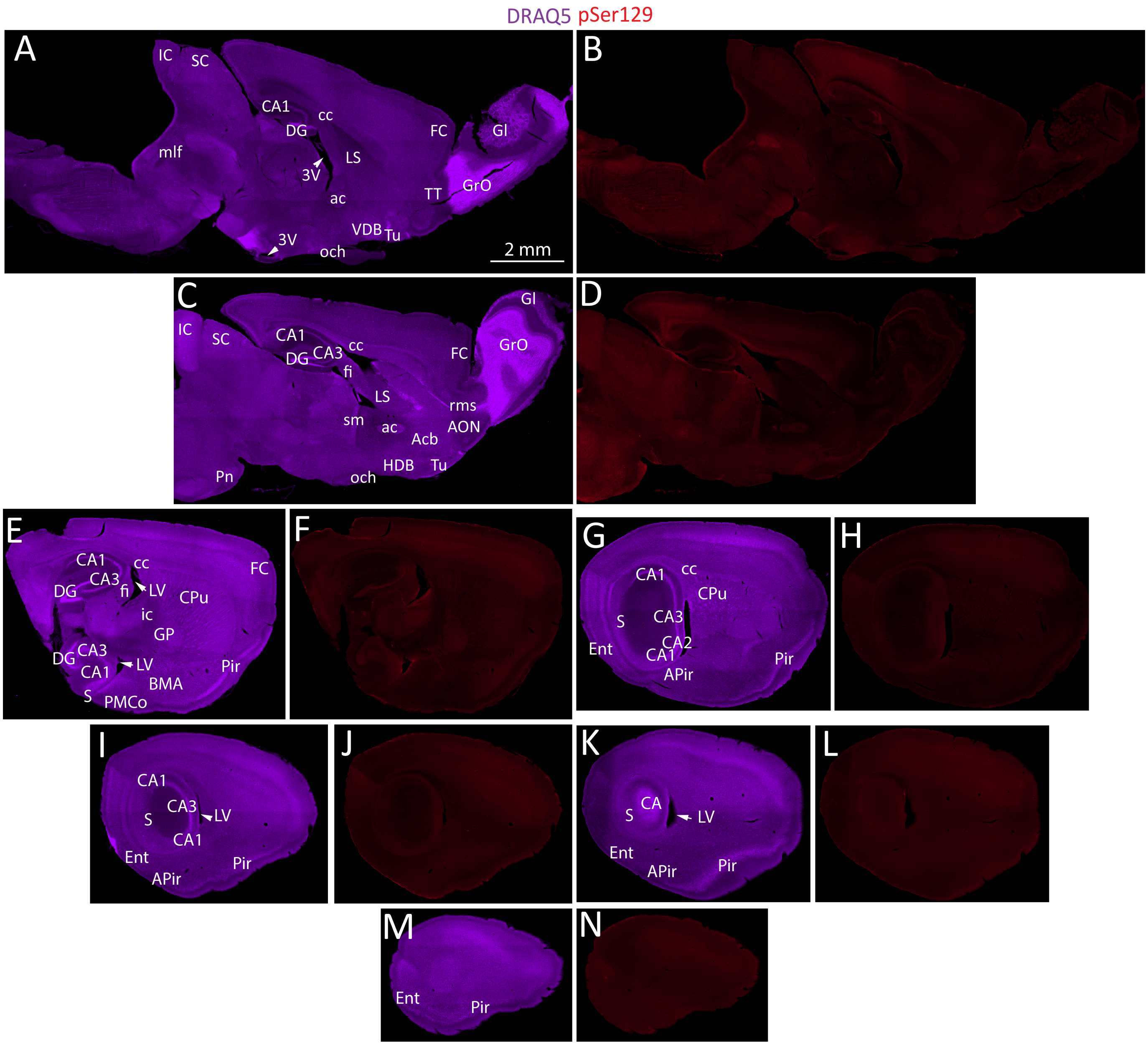


**Fig.S8** Mice were infused with phosphate-buffered saline (PBS) into the OB/AON. Three months later, sagittal brain sections were collected and stained for DRAQ5 (**A, C, E, G, I, K, M**) and pSer129 (**B, D, F, H, J, L, N**). Panels A-B, E-F, I-J, M-N illustrate animals infused with PBS at 17 months of age and sacrificed at 20 months. The remaining panels illustrate animals that were infused at 5 months of age and sacrificed at 8 months. The exposure times for all the microscopy in this study was purposefully long so that all the background staining (particularly of fiber tracts) and the cytoarchitectonic boundaries could be properly discerned. Abbreviations in **Table S1**

# References


Beach, T.G., White, C.L., 3rd, Hladik, C.L., Sabbagh, M.N., Connor, D.J., Shill, H.A., Sue, L.I., Sasse, J., Bachalakuri, J., Henry-Watson, J., *et al.* (2009). Olfactory bulb alpha-synucleinopathy has high specificity and sensitivity for Lewy body disorders. Acta Neuropathol *117*, 169-174.

Braak, H., Del Tredici, K., Rub, U., de Vos, R.A., Jansen Steur, E.N., and Braak, E. (2003). Staging of brain pathology related to sporadic Parkinson's disease. Neurobiol Aging *24*, 197-211.

Daniel, S.E., and Hawkes, C.H. (1992). Preliminary diagnosis of Parkinson's disease by olfactory bulb pathology. Lancet *340*, 186.

Dickson, D.W., Schmidt, M.L., Lee, V.M., Zhao, M.L., Yen, S.H., and Trojanowski, J.Q. (1994). Immunoreactivity profile of hippocampal CA2/3 neurites in diffuse Lewy body disease. Acta Neuropathol *87*, 269-276.

Flores-Cuadrado, A., Ubeda-Banon, I., Saiz-Sanchez, D., de la Rosa-Prieto, C., and Martinez-Marcos, A. (2016). Hippocampal alpha-synuclein and interneurons in Parkinson's disease: Data from human and mouse models. Mov Disord.

Franklin, K.B.J., and Paxinos, G. (2013). Paxinos and Franklin's The mouse brain in stereotaxic coordinates, Fourth edition. edn (Amsterdam: Academic Press, an imprint of Elsevier).

Jiao, Y., Sun, Z., Lee, T., Fusco, F.R., Kimble, T.D., Meade, C.A., Cuthbertson, S., and Reiner, A. (1999). A simple and sensitive antigen retrieval method for free-floating and slide-mounted tissue sections. J Neurosci Methods *93*, 149-162.

Luk, K.C., Kehm, V., Carroll, J., Zhang, B., O'Brien, P., Trojanowski, J.Q., and Lee, V.M. (2012a). Pathological alpha-synuclein transmission initiates Parkinson-like neurodegeneration in nontransgenic mice. Science *338*, 949-953.

Luk, K.C., Kehm, V.M., Zhang, B., O'Brien, P., Trojanowski, J.Q., and Lee, V.M. (2012b). Intracerebral inoculation of pathological alpha-synuclein initiates a rapidly progressive neurodegenerative alpha-synucleinopathy in mice. The Journal of experimental medicine *209*, 975-986.

Luk, K.C., Song, C., O'Brien, P., Stieber, A., Branch, J.R., Brunden, K.R., Trojanowski, J.Q., and Lee, V.M. (2009). Exogenous alpha-synuclein fibrils seed the formation of Lewy body-like intracellular inclusions in cultured cells. Proceedings of the National Academy of Sciences of the United States of America *106*, 20051-20056.

Paumier, K.L., Luk, K.C., Manfredsson, F.P., Kanaan, N.M., Lipton, J.W., Collier, T.J., Steece-Collier, K., Kemp, C.J., Celano, S., Schulz, E., *et al.* (2015). Intrastriatal injection of pre-formed mouse alpha-synuclein fibrils into rats triggers alpha-synuclein pathology and bilateral nigrostriatal degeneration. Neurobiol Dis *82*, 185-199.

Rey, N.L., Petit, G.H., Bousset, L., Melki, R., and Brundin, P. (2013). Transfer of human alpha-synuclein from the olfactory bulb to interconnected brain regions in mice. Acta neuropathologica *126*, 555-573.

Saper, C.B. (2005). An open letter to our readers on the use of antibodies. J Comp Neurol *493*, 477-478.

Schmued, L.C., and Fallon, J.H. (1986). Fluoro-Gold: a new fluorescent retrograde axonal tracer with numerous unique properties. Brain Res *377*, 147-154.

Swanson, L.W. (2004). Brain maps III : structure of the rat brain : an atlas with printed and electronic templates for data, models, and schematics, 3rd rev. edn (Amsterdam ; Boston: Elsevier, Academic Press).

Ubeda-Banon, I., Saiz-Sanchez, D., de la Rosa-Prieto, C., Argandona-Palacios, L., Garcia-Munozguren, S., and Martinez-Marcos, A. (2010). alpha-Synucleinopathy in the human olfactory system in Parkinson's disease: involvement of calcium-binding protein- and substance P-positive cells. Acta Neuropathol *119*, 723-735.

Volpicelli-Daley, L.A., Luk, K.C., and Lee, V.M. (2014). Addition of exogenous alpha-synuclein preformed fibrils to primary neuronal cultures to seed recruitment of endogenous alpha-synuclein to Lewy body and Lewy neurite-like aggregates. Nat Protoc *9*, 2135-2146.

Volpicelli-Daley, L.A., Luk, K.C., Patel, T.P., Tanik, S.A., Riddle, D.M., Stieber, A., Meaney, D.F., Trojanowski, J.Q., and Lee, V.M. (2011). Exogenous alpha-synuclein fibrils induce Lewy body pathology leading to synaptic dysfunction and neuron death. Neuron *72*, 57-71.

Watson, R.E., Jr., Wiegand, S.J., Clough, R.W., and Hoffman, G.E. (1986). Use of cryoprotectant to maintain long-term peptide immunoreactivity and tissue morphology. Peptides *7*, 155-159.

Waxman, E.A., and Giasson, B.I. (2008). Specificity and regulation of casein kinase-mediated phosphorylation of alpha-synuclein. Journal of neuropathology and experimental neurology *67*, 402-416.

Wessendorf, M.W. (1991). Fluoro-Gold: composition, and mechanism of uptake. Brain Res *553*, 135-148.
